# Flavonoids as Immunoregulators: Molecular Mechanisms in Regulating Immune Cells and Their Therapeutic Applications in Inflammatory Diseases

**DOI:** 10.3389/fimmu.2025.1703672

**Published:** 2025-10-31

**Authors:** Yingshu Liu, Ao Jiao

**Affiliations:** ^1^ Department of Laboratory Medicine, Shengjing Hospital of China Medical University, Shenyang, Liaoning, China; ^2^ Liaoning Clinical Research Center for Laboratory Medicine, Shenyang, Liaoning, China; ^3^ Department of Hepatopancreatobiliary Surgery, Cancer Hospital of China Medical University, Shenyang, Liaoning, China; ^4^ Department of Hepatopancreatobiliary Surgery, Cancer Hospital of Dalian University of Technology, Shenyang, Liaoning, China; ^5^ Pancreatic Disease Center, Liaoning Cancer Hospital & Institute, Shenyang, Liaoning, China

**Keywords:** flavonoids, immunoregulators, immune cells, inflammatory diseases, molecular mechanism

## Abstract

Flavonoids are a vital class of dietary polyphenolic compounds that have attracted considerable attention owing to their powerful immunoregulatory and anti-inflammatory effects. This review summarizes recent advances in understanding the role of flavonoids in regulating immune cells and their therapeutic application in inflammatory diseases. We present an overview of the definition, classification, and dietary sources of flavonoids and detail their regulatory effects on multiple key immune cells, therapeutic potential of flavonoids in various inflammatory diseases, as well as discuss strategies to improve their bioavailability and targeting. Despite the promising immunoregulatory properties of flavonoids, their clinical utilization is impeded by issues such as low bioavailability, considerable interindividual variability, and the absence of high-quality randomized controlled trials. Future research needs to focus on elucidating the precise mechanisms of flavonoids, optimizing their pharmacokinetic properties, and conducting more standardized clinical trials to facilitate the transformation of these natural compounds into standardized immunomodulatory therapeutic agents.

## 1 Introduction

Inflammatory diseases have emerged as a significant global public health concern, encompassing inflammatory bowel disease (IBD), rheumatoid arthritis (RA), metabolic inflammatory disorders, neuroinflammatory conditions, respiratory inflammatory diseases, etc. Global data indicate that the prevalence of IBD ranges from 0.5% to 1% in Western nations and is increasing in emerging industrialized countries and may reach 1% in numerous regions by 2030 (1). RA affects approximately 0.5% to 1% of people worldwide (2). Metabolic inflammatory disorders, an inflammatory condition resulting from obesity and type 2 diabetes, collectively pose a substantial threat to human health (3). The shared pathological foundation of these diseases is immune system dysfunction, resulting in the overproduction of inflammatory mediators, inappropriate activation of immune cells, and ultimately causing tissue damage (4). The persistent presence of inflammation and the disruption of immune homeostasis are the primary mechanisms contributing to the chronic progression and treatment difficulties of these diseases.

Contemporary therapeutic approaches for inflammatory diseases include nonsteroidal anti-inflammatory drugs (NSAIDs), corticosteroids, disease-modifying antirheumatic drugs (DMARDs), and biologics (5). Although these therapies have effectively managed symptoms and decelerated disease progression, long-term use is often associated with severe adverse effects, including increased infection risk, osteoporosis, metabolic disorders, and the likelihood of malignant tumors (6, 7). Additionally, a considerable proportion of patients exhibit poor response to existing treatments or gradually lose responsiveness (8). Therefore, the development of secure and effective novel anti-inflammatory drugs or supplementary treatment strategies has become an urgent clinical need. In this context, flavonoid compounds derived from natural plants have attracted widespread attention owing to their broad immunomodulatory effects and minimal toxicity (9).

Flavonoids are a class of polyphenolic compounds characterized by a C6-C3-C6 fundamental structure, capable of regulating immune cell function through various mechanisms. However, their clinical application faces challenges. Their low bioavailability, short half-life in the body, and complex metabolic conversion make it difficult to achieve effective therapeutic concentrations (10). Furthermore, the immunoregulatory effects of flavonoids display dose-dependent and cell-specific characteristics, potentially exhibiting bidirectional regulatory effects, thereby complicating their clinical application (11, 12). Recent years have witnessed advancements in the research concerning the immunomodulatory effects of flavonoids. Advanced high-sensitivity analytical techniques enable researchers to identify flavonoids and their metabolites *in vivo* even when concentrations are extremely low, facilitating a more precise evaluation of their bioavailability (13). Studies on structure-activity relationships have identified crucial active domains, guiding the development of more efficient flavonoid derivatives (14). Randomized controlled trials (RCTs) and non-RCT clinical trials have proved the therapeutic value of flavonoids in various diseases (15, 16).

Current reviews mainly focus on the impacts of particular subclasses of flavonoids or their effects within particular disease contexts, while a holistic perspective is absent. Many dietary components contain multiple flavonoids. Thus, traditional Chinese medicine, Tibetan medicine, and other ethnic medicines frequently consist of compound formulations containing diverse flavonoids. Furthermore, inflammatory diseases frequently coexist. It is of great significance to understand in detail the abundance of a specific flavonoid compound in a certain food or medicine and its therapeutic effects, mainly in different immune cells and various inflammatory diseases. We aimed to summarize how flavonoids regulate immune cells and their applications in the treatment of inflammatory diseases. By integrating the latest research evidence, we will provide a comprehensive understanding of the immunoregulatory capabilities of flavonoids and their rational application in the clinical treatment of inflammatory diseases.

## 2 Definition, classification, and dietary sources of flavonoids

Flavonoids are a category of polyphenolic compounds that are commonly present in consumable plants and foods, including fruits, vegetables, grains, and nuts, primarily present in the form of β-glycosides (17). The fundamental structure of these compounds is constituted by a 15-carbon framework, which is comprised of two benzene rings (A and B) linked through a heterocyclic furan ring (C) (18). Currently, the identification of flavonoid compounds has exceeded 10, 000, with the figure continuing to increase (19). Flavonoids are classified according to their chemical structure into anthocyanins, flavan-3-ols, flavonols, flavones, flavanones, isoflavones, etc (**Table 1**). The physiological effects of flavonoids include anti-inflammatory properties, immune modulation, antioxidant activity, and regulation of gut microbiota (9, 20–22).

**Table 1:** Common classifications of flavonoids and dietary sources.

Classification	Common compounds		Chemical structure and molecular formula	Dietary sources	Mean* (mg/100g)	Reference
Anthocyanidins	Cyanidin		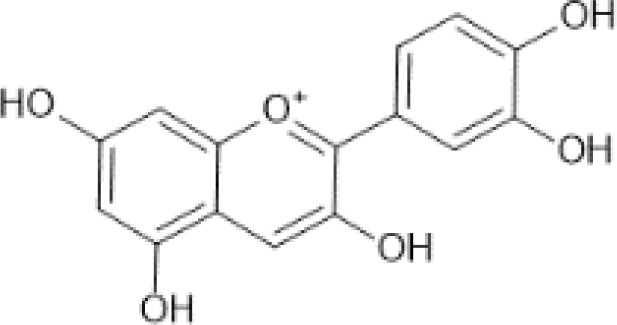 C_15_H_11_O_6_^+^	Peppers, tasmanian	752.68	(29–31)
				Raspberries, black	669.01	
				Plum, Illawara, raw (Podocarpus elatus)	555.72	
				Elderberries, raw (Sambucus spp.)	485.26	
				Juice concentrate, elderberry	411.40	
	Delphinidin		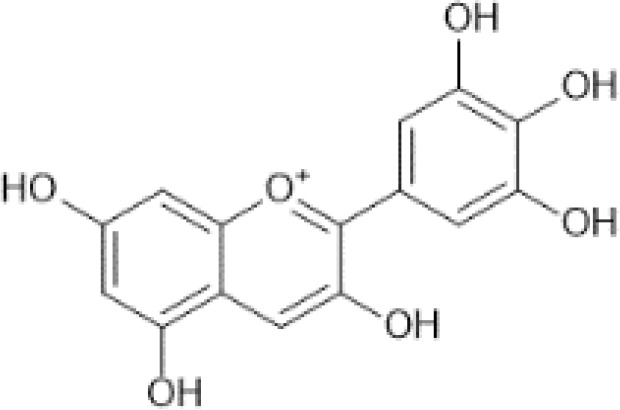 C_15_H_11_O_7_^+^	Juice concentrate, black currant	201.28	(29, 32, 33)
				Bilberry, raw	97.59	
				Cowpeas, black seed cultivar, mature seeds, raw (Vigna unguiculata Subsp. Sinensis)	94.60	
				Currants, european black, raw (Ribes nigrum)	89.62	
				Eggplant, raw (Solanum melongena)	85.69	
	Malvidin		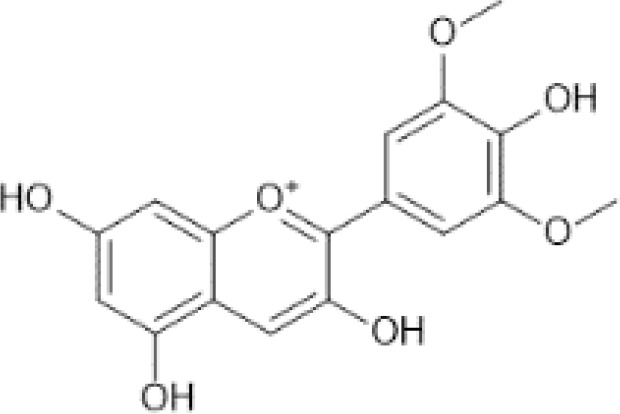 C_17_H_15_O_7_^+^	Alcoholic beverage, wine, table, red, Syrah or Shiraz	121.65	(29, 34, 35)
				Alcoholic beverage, wine, dessert, sweet	94.83	
				Blueberries, cultivated (highbush), raw (Vaccinium spp.)	67.59	
				Blueberries, rabbiteye, raw (Vaccinium spp.)	63.45	
				Blueberries, wild (lowbush), raw (Vaccinium spp.)	57.16	
	Pelargonidin		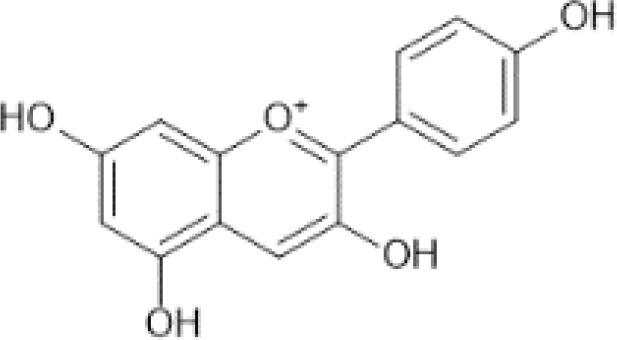 C_15_H_11_O_5_^+^	Radishes, raw (Raphanus sativus)	63.13	(29, 36, 37)
				Strawberries, raw (Fragaria X ananassa)	24.85	
				Strawberries, frozen, unsweetened	19.32	
				Raspberries, black	16.69	
				Juice, strawberry	11.79	
	Peonidin		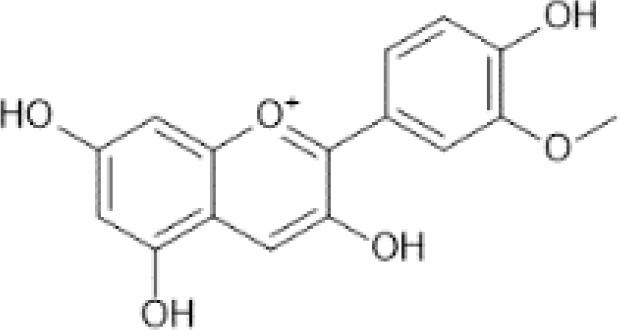 C_16_H_13_O_6_^+^	Cranberries, raw (Vaccinium macrocarpon)	30.54	(29, 38, 39)
				Bilberry, raw	20.45	
				Blueberries, cultivated (highbush), raw (Vaccinium spp.)	20.29	
				Blueberries, rabbiteye, raw (Vaccinium spp.)	15.90	
				Cowpeas, black seed cultivar, mature seeds, raw (Vigna unguiculata Subsp. Sinensis)	11.07	
	Petunidin		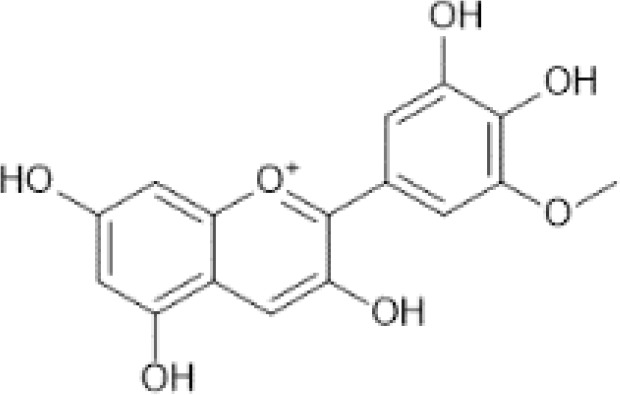 C_16_H_13_O_7_^+^	Guajiru (coco-plum), raw	55.72	(29, 35, 40)
				Bilberry, raw	42.69	
				Blueberries, rabbiteye, raw (Vaccinium spp.)	36.25	
				Blueberries, cultivated (highbush), raw (Vaccinium spp.)	31.53	
				Cowpeas, black seed cultivar, mature seeds, raw (Vigna unguiculata Subsp. Sinensis)	27.82	
Flavan-3-ols	Monomers (Catechins) and their gallate derivatives (41)	(+)-Catechin	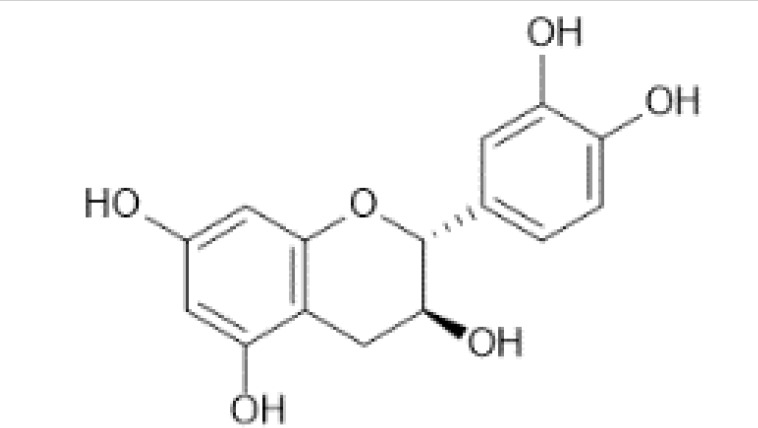 C_15_H_14_O_6_	Blueberries, rabbiteye, raw (Vaccinium spp.)	98.47	(29, 42, 43)
				Cacao beans	88.45	
				Grape seeds, raw	74.63	
				Tea, green, large leaf, Quingmao, brewed	67.60	
				Cocoa, dry powder, unsweetened	64.82	
		(–)-Epicatechin	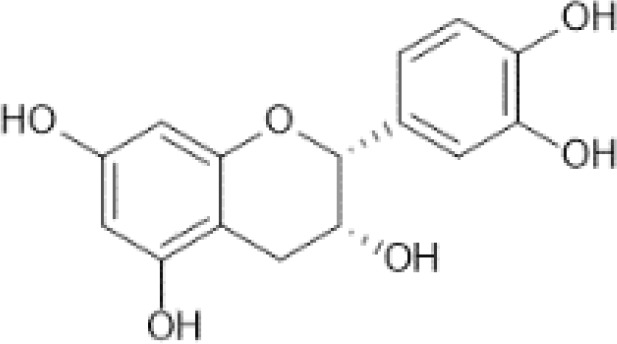 C_15_H_14_O_6_	Cocoa, dry powder, unsweetened	196.43	(29, 44, 45)
				Baking chocolate, unsweetened, squares	141.83	
				Cacao beans	99.18	
				Grape seeds, raw	93.31	
				Candies, chocolate, dark	84.40	
		(–)-Epigallocatechin	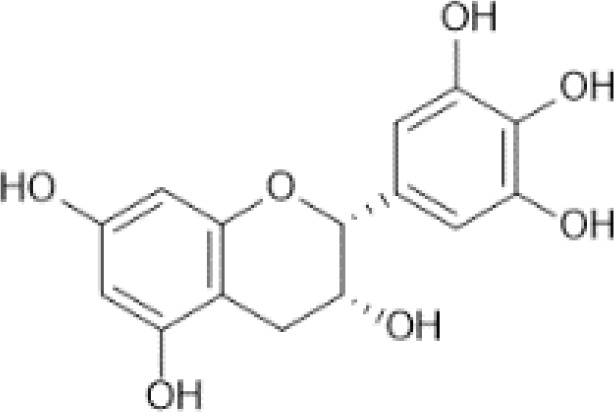 C_15_H_14_O_7_	Cacao beans	156.67	(29, 46, 47)
				Tea, green, large leaf, Quingmao, brewed	19.80	
				Tea, white, brewed	18.65	
				Tea, green, brewed, decaffeinated	16.02	
				Broadbeans, immature seeds, raw (Vicia faba)	15.47	
		(+)-Gallocatechin	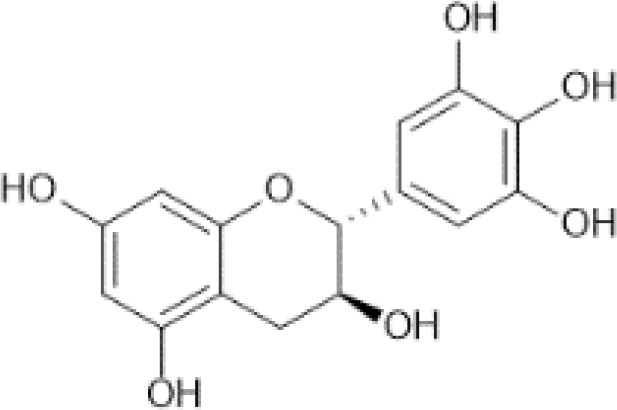 C_15_H_14_O_7_	Cacao beans	8262.00	(29, 48, 49)
				Marrowfat pea, canned, drained solids	4.33	
				Broadbeans, immature seeds, raw (Vicia faba)	4.15	
				Strawberry tree fruit (arbutus), raw	1.60	
				Currants, red, raw	1.28	
	Dimers and Polymers (50)	Proanthocyanidins	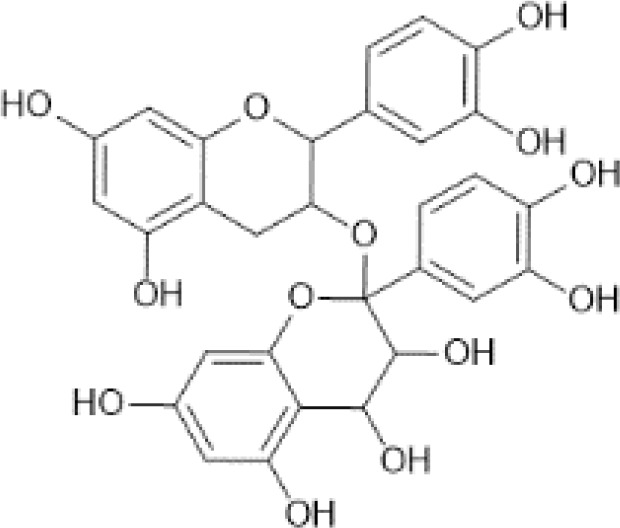 C_30_H_26_O_13_	Sorghum bran, Sumac (Sorghum bicolor)	2927.64	(51–53)
				Spices, cinnamon, ground (Cinnamomum aromaticum)	2508.78	
				Cocoa, dry powder, unsweetened	2435.11	
				Cacao beans (Theobroma cacoa)	1568.49	
				Sorghum grain (Sorghum bicolor)	1346.28	
	Theaflavin		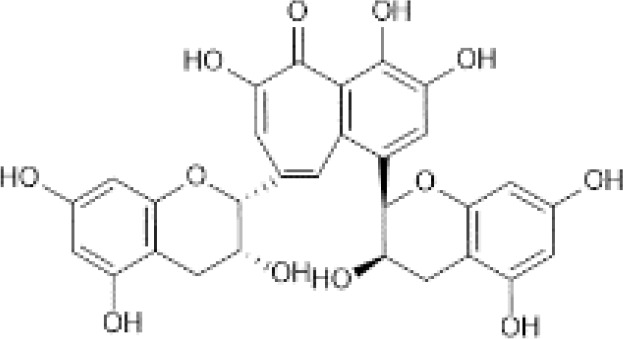 C_29_H_24_O_12_	Tea, black, brewed, prepared with tap water	1.58	(29, 54)
				Tea, black, brewed, prepared with tap water, decaffeinated	0.35	
				Tea, green, brewed, decaffeinated	0.12	
				Tea, black, ready-to-drink, plain and flavored	0.05	
				Tea, green, brewed, flavored	0.02	
	Thearubigins (41)		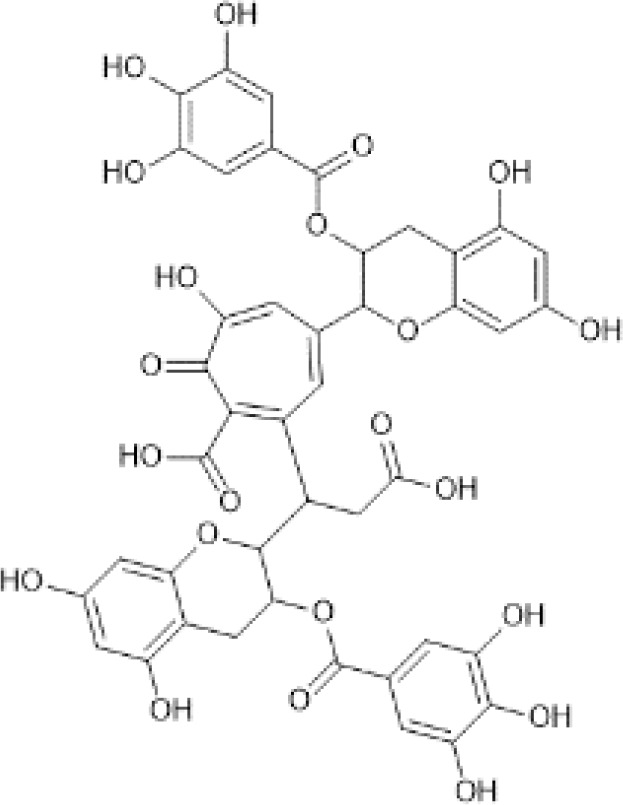 C_43_H_34_O_22_	Tea, black, brewed, prepared with tap water	81.30	(29, 55)
				Tea, black, brewed, prepared with tap water, decaffeinated	49.03	
				Tea, instant, sweetened with sugar, plain and flavored, prepared	27.95	
				Tea, black, ready-to-drink, plain and flavored	25.49	
				Tea, instant, unsweetened, powder, prepared	23.65	
Flavonols (56)	Quercetin		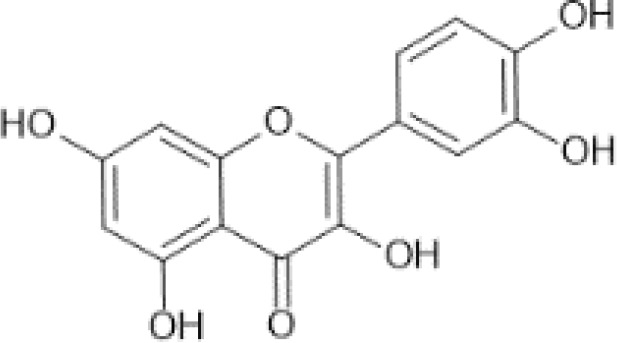 C_15_H_10_O_7_	Capers, raw	233.84	(29, 57)
				Capers, canned (Capparis spinosa)	172.55	
				Lovage, leaves, raw	170.00	
				Juice concentrate, elderberry	108.16	
				Dock, raw (Rumex spp.)	86.20	
	Kaempferol		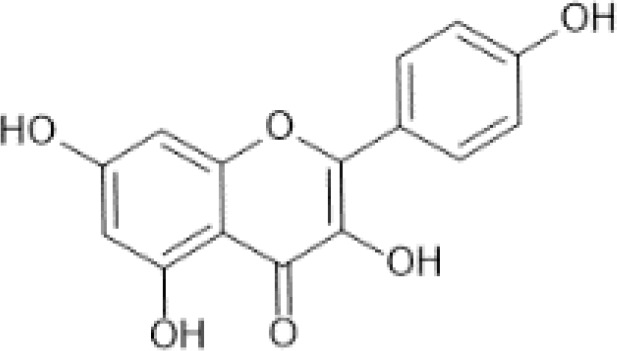 C_15_H_10_O_6_	Capers, raw	259.19	(29, 58, 59)
				Spices, saffron (Crocus sativus)	205.48	
				Capers, canned (Capparis spinosa)	131.34	
				Kale, raw (Brassica oleracea (Acephala Group))	46.80	
				Mustard greens, raw (Brassica juncea)	38.30	
	Myricetin		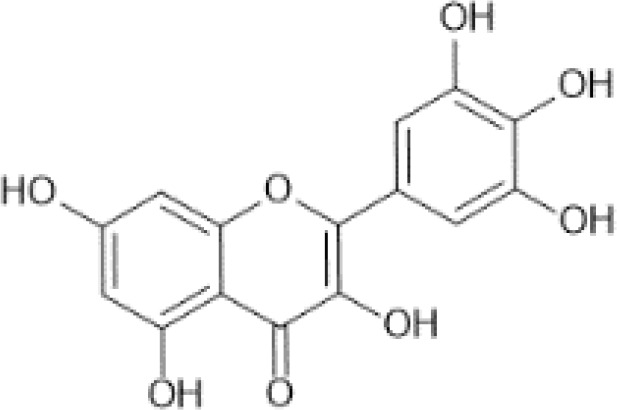 C_15_H_10_O_8_	Carob kibbles	47.74	(29, 60, 61)
				Juice concentrate, black currant	20.85	
				Fennel, leaves, raw	19.80	
				Parsley, fresh (Petroselinum crispum)	14.84	
				Carob kibbles	11.67	
	Isorhamnetin		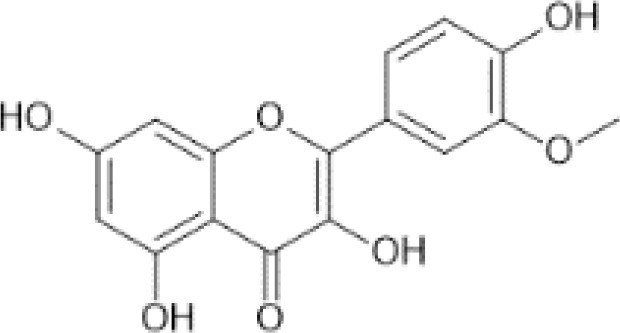 C_16_H_12_O_7_	Spices, parsley, dried (Petroselinum crispum)	331.24	(29, 62, 63)
				Dill weed, fresh (Anethum graveolens)	43.50	
				Sea buckthorn berry, raw	38.29	
				Kale, raw (Brassica oleracea (Acephala Group))	23.60	
				Mustard greens, raw (Brassica juncea)	16.20	
Flavones (64)	Apigenin		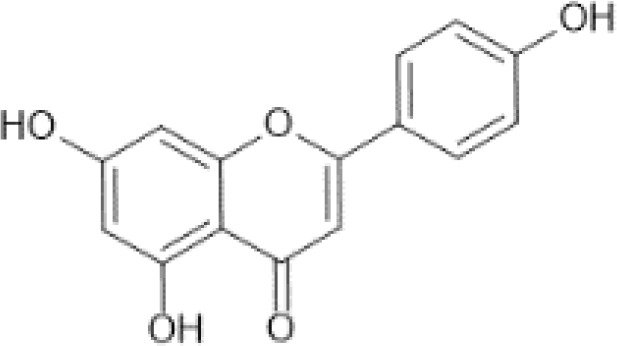 C_15_H_10_O_5_	Spices, parsley, dried (Petroselinum crispum)	4503.50	(29, 65, 66)
				Parsley, fresh (Petroselinum crispum)	215.46	
				Spices, celery seed (Apium graveolens)	78.65	
				Vinespinach, (basella), raw (Basella alba)	62.20	
				Celery, Chinese, raw	24.02	
	Luteolin		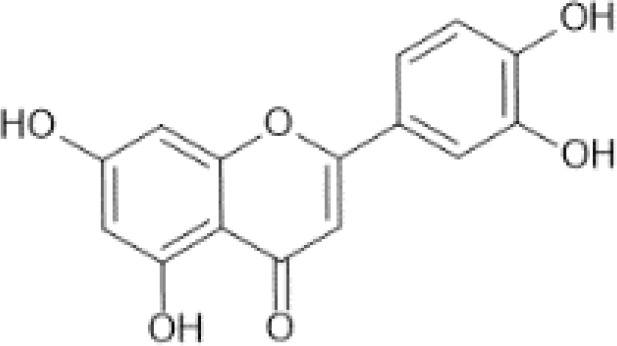 C_15_H_10_O_6_	Oregano, Mexican, dried	1028.75	(29, 67, 68)
				Spices, celery seed (Apium graveolens)	762.40	
				Juniper berries, ripe (Juniperus communis)	69.05	
				Juniper berries, green, unripe (Juniperus communis)	51.40	
				Thyme, fresh (Thymus vulgaris)	45.25	
Flavanones (69)	Eriodictyol		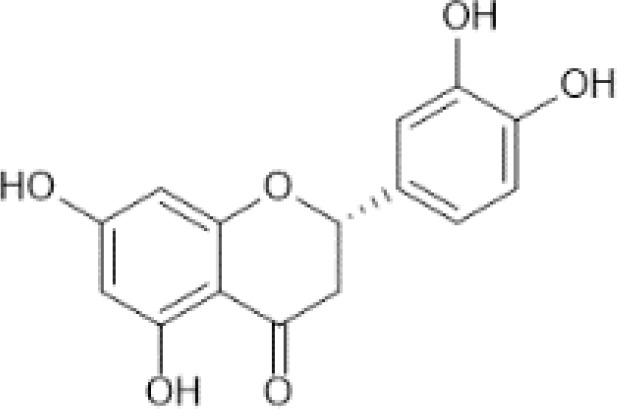 C_15_H_12_O_6_	Oregano, Mexican, dried	85.33	(29, 70)
				Peppermint, fresh (Mentha x piperita L. nothosubsp. piperita)	30.92	
				Lemons, raw, without peel (Citrus limon)	21.36	
				Juice, sour orange	14.54	
				Juice, lemon, canned or bottled	10.56	
	Hesperetin		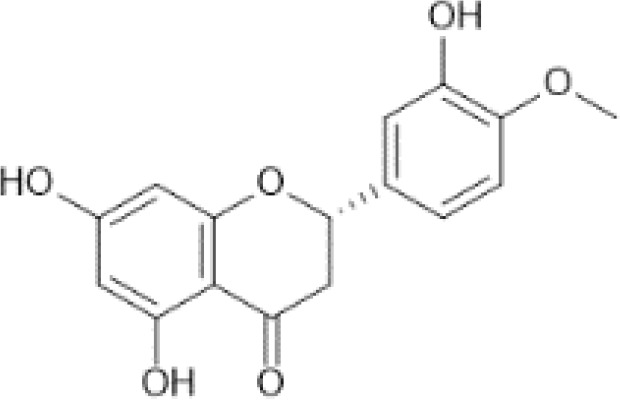 C_16_H_14_O_6_	Juice, tangelo	74.89	(29, 71)
				Limes, raw (Citrus latifolia)	43.00	
				Yuzu, raw	28.73	
				Lemons, raw, without peel (Citrus limon)	27.90	
				Oranges, raw, all commercial varieties (Citrus sinensis)	27.25	
	Naringenin		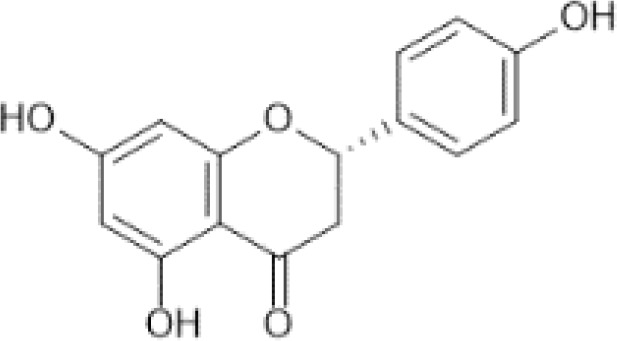 C_15_H_12_O_5_	Oregano, Mexican, dried	372.00	(29, 70, 72)
				Kumquats, raw (Fortunella spp.)	57.39	
				Grapefruit, raw (not specified as to color) (Citrus paradisi)	53.00	
				Juice, tangelo	42.51	
				Grapefruit, raw, pink and red, all areas (Citrus paradisi)	32.64	
Isoflavones (73)	Formononetin		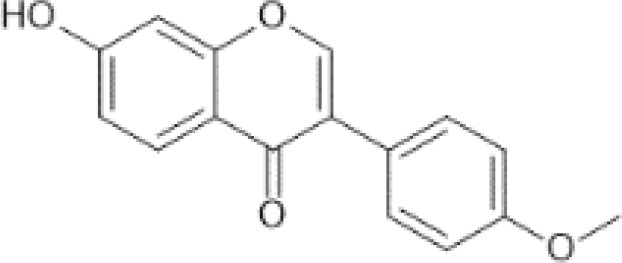 C_16_H_12_O_4_	Red clover	833.00	(74, 75)
				Soybeans, mature seeds, raw	8.46	
				Clover sprouts, raw	3.15	
				Alfalfa seeds, sprouted, raw	1.43	
				Beans, pink, mature seeds, raw	1.05	
	Genistein		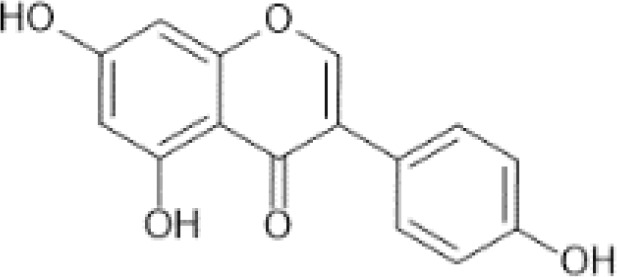 C_15_H_10_O_5_	Soy meal, defatted, raw	114.71	(74, 76)
				Soymilk skin or film (Foo jook or yuba), raw	101.40	
				Soy flour, full-fat, raw	98.77	
				Soybeans, flakes, defatted	91.22	
				Soy flour (textured)	89.42	
	Daidzein		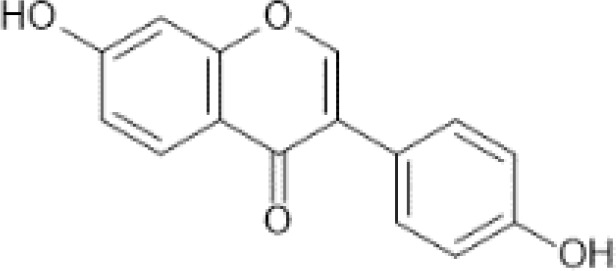 C_15_H_10_O_4_	Soy flour, full-fat, roasted	89.46	(74, 77)
				Soy meal, defatted, raw	80.77	
				Soymilk skin or film (Foo jook or yuba), raw	80.03	
				Soybeans, mature seeds, raw (Korea)	78.86	
				Soy flour, full-fat, raw	72.92	
	Coumestrol		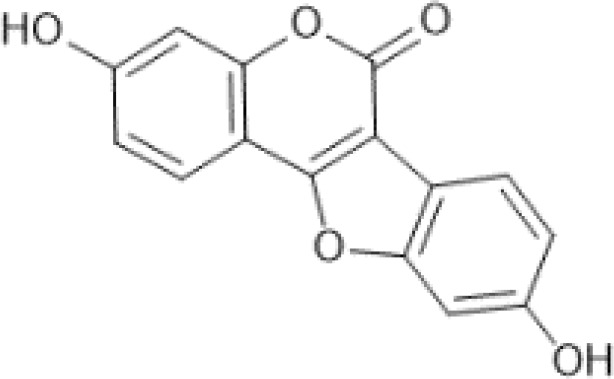 C_15_H_8_O_5_	Red clover	1322.00	(74, 78, 79)
				Clover sprouts, raw	14.08	
				Kala chana, mature seeds, raw	6.13	
				Beans, pinto, mature seeds, raw	1.80	
				Alfalfa seeds, sprouted, raw	1.60	
	Glycitein		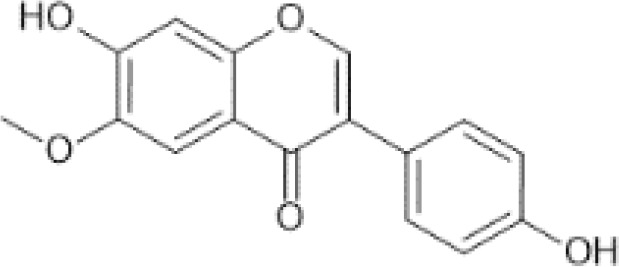 C_16_H_12_O_5_	Soybeans, mature seeds, raw (Europe)	22.37	(74, 80)
				Soy flour (textured)	20.02	
				Soybeans, mature seeds, raw (Korea)	18.76	
				Soybeans, mature seeds, raw (Australia)	17.12	
				Soy flour, full-fat, roasted	16.40	
	Biochanin A		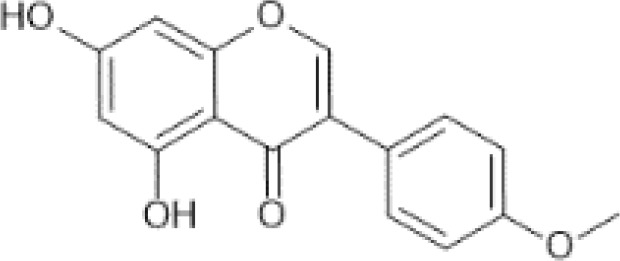 C_16_H_12_O_5_	Chickpeas (garbanzo beans, bengal gram), mature seeds, raw	1.54	(74, 81, 82)
				Kala chana, mature seeds, raw	1.26	
				Beans, great northern, mature seeds, raw	0.60	
				Clover sprouts, raw	0.59	
				Cowpeas, common, (blackeyes, crowder, southern), mature seeds, raw	0.58	

*According to USDA database, mean values were reported as mg/100g of fresh weight of edible portion of food. For beverages, mean values reported on a liquid basis (mg/mL) were converted to a weight basis (mg/100 g) using the corresponding specific gravities. Tea was prepared as infusions (1% infusion = 1g tea leaves/100ml boiling water). Mean values of tea were reported as mg/100g of tea infusions (mg per 100 ml brewed tea prepared from 1g of dry tea leaves).

The absorption process of flavonoids is complicated and affected by their chemical structure, solubility, membrane permeability, and the metabolism of gut microbiota. Different classes of flavonoids possess distinct structural features, resulting in variations in their metabolic pathways and bioavailability within the body (23). However, the bioavailability of most flavonoids is limited due to their prevalence in glycoside forms, making them difficult for the digestive system to absorb (24). Flavonoid glycosides need to be hydrolyzed into aglycone forms so that they can pass through intestinal epithelial cells effectively (25). Additionally, some flavonoids are quickly broken down and become inactive by enzymes in the intestines and liver after ingestion, which, to some extent, reduces the amount of active compounds that reach the bloodstream (23). Recent research has developed drug delivery systems by adding flavonoids into carriers, such as nanoemulsions, metal-organic frameworks, and alginate-chitosan microspheres. These systems provided new approaches to improve the absorption and bioavailability of flavonoids (26–28).

## 3 The regulatory effect of flavonoids on immune cells

### 3.1 Macrophages

Macrophages are a type of innate immune cells with phagocytic and antigen-presenting functions, playing a crucial role in various diseases (83). Activated macrophages are generally categorized into two kinds: M1 macrophages, which participate in pro-inflammatory responses, and M2 macrophages, which engage in anti-inflammatory responses (84). In inflammatory conditions, a key therapeutic strategy is to promote the differentiation of pro-inflammatory M1 macrophages into anti-inflammatory M2 macrophages. Flavonoids have been demonstrated to have the capacity to ameliorate inflammatory diseases by suppressing M1 polarization and enhancing M2 polarization in macrophages. Quercetin, puerarin, and luteolin can increase M2 markers (e.g., Arg1 and IL-10) via the PI3K/Akt, PPARγ, or AMPK pathways, thereby facilitating macrophage polarization towards M2 (85–87). In contrast, baicalin, Allium cepa L. peel extract, apigenin, and hesperidin can prevent macrophage polarization toward the M1 phenotype by blocking the JAK/STAT, MyD88/NF-κB, MAPK, direct kinase targets (IRAK4), or Jagged1/Notch1 pathways (88–91). Flavonoids have also been proven to influence macrophage polarization by regulating metabolic reprogramming. Phellodendrin has been shown to inhibit HIF-1α-regulated macrophage PI3K/Akt and glycolysis, restoring the M1/M2 macrophage balance in periodontitis, thereby reducing the pro-inflammatory effects and immune dysfunction caused by overactivated M1 macrophages (92).

NLRP3 inflammasome is a multiprotein complex existing in macrophages linked to a number of chronic inflammatory diseases. It can initiate the death of inflammatory cells and the release of the pro-inflammatory cytokines IL-1β and IL-18 (93). It also serves as the main target for flavonoid compounds in regulating macrophages. Quercetin, luteolin, and apigenin can prevent NLRP3 inflammasome assembly, activate caspase-1, and cleave Gasdermin D (GSDMD) through reactive oxygen species (ROS) scavenging or Nrf2/HO-1 activation, thereby decreasing IL-1β secretion, inhibiting macrophage pyroptosis, and suppressing macrophage-driven inflammation (94–96).

New delivery systems have been developed to improve the effectiveness of flavonoid compounds targeting macrophages. Macrophage membrane-modified baicalin liposomes (MM-BA-LP), compared to conventional baicalin liposomes (BA-LP), offer better cerebral targeting, improved pharmacokinetics, and longer retention in the bloodstream (97). Zhang et al. constructed M2M@BANPs by loading baicalin onto poly (lactic-co-glycolic acid) nanoparticles and encapsulating them within M2 macrophage membranes. Under the coating of M2M, M2M@BANPs can effectively target ischemic brain tissue and accumulate in microglia and neurons. This reprograms microglia from M1 to M2, significantly impacting therapeutic effects in ischemic stroke (98).

Although most flavonoids show their anti-inflammatory properties, certain studies have reported their pro-inflammatory effects. In the tumor microenvironment, vitexin has been observed to promote macrophage polarization towards the M1 phenotype via the VDR/PBLD pathway, thereby enhancing anticancer activity (99). In the mouse peritoneal infection model, intraperitoneal injection of epigallocatechin gallate (EGCG) significantly increased the quantity of monocytes/macrophages in both the peritoneal cavity and peripheral blood. EGCG can also induce pro-inflammatory responses in macrophages and promote phagocyte migration in co-culture systems containing macrophages, indicating that EGCG is an effective agent for preventing bacterial infections (100). The divergent regulatory effects of flavonoids on macrophage anti-inflammatory and pro-inflammatory responses indicate that their regulation is contingent upon environmental factors.

### 3.2 Neutrophil

Neutrophils are the most abundant type of leukocytes in the circulatory system. As effector cells of the innate immune system, they play a key role in the pathophysiological processes of inflammatory and autoimmune disorders (101, 102). Recent studies have shown that, beyond eliminating pathogens through traditional phagocytosis, neutrophils participate in anti-infective defense and inflammatory responses by releasing neutrophil extracellular traps (NETs), secreting inflammatory mediators (e.g., cytokines, chemokines, and lipid mediators), and producing ROS (103, 104). However, excessive activation of neutrophils can result in tissue damage and the development of inflammatory diseases. Flavonoids have shown anti-inflammatory effects through the regulation of neutrophils.

NETs are network-like structures composed of chromatin and granular proteins released by activated neutrophils. Although these structures contribute to defense mechanisms by capturing pathogens, the excessive or abnormal NETs formation can contribute to the pathogenesis of inflammatory and autoimmune disorders. NETs can amplify localized inflammation and induce tissue damage. Accumulating evidence indicates that flavonoids exert anti-inflammatory effects by regulating neutrophil function and inhibiting NETs formation through multiple signaling pathways. For instance, quercetin inhibits NETs formation by targeting the P2X7R/P38MAPK/NOX2 signaling pathway (105), thereby alleviating oxidative stress and inflammatory responses in neutrophils. Astilbin suppresses NETs formation by regulating the purinergic P2Y6 receptor and the IL-8/CXCR2 pathway (106). Dihydromyricetin blocks NETs formation through the HIF-1α/VEGFA pathway (107). Additionally, calycosin decreases NETs formation and neutrophil-mediated inflammation by acting on the TLR4/NF-κB pathway (108). Overall, these results show that flavonoids are effective inhibitors of NETs, potentially assisting in the reduction of inflammation and the development of autoimmune conditions.

ROS serve as critical effector molecules for neutrophil killing of pathogens, but excessive ROS may cause tissue damage. Research shows that luteolin can inhibit the production of ROS by regulating neutrophils, a process aided by the suppression of Raf1 activity (109). Flavonoids are capable of inhibiting overall ROS production; however, their effectiveness varies among different types. The condensation product of taxifolin with glyoxylic acid (DfTf) significantly decreases extracellular ROS levels with no effect on intracellular ROS. In contrast, naringin is more effective at inhibiting intracellular ROS (110). The role of ROS, intracellularly and extracellularly, varies in health and disease. Intracellular ROS has been shown to suppress IL-1β expression, thereby limiting neutrophil recruitment. In contrast, extracellular ROS appears to enhance IL-1β expression, promoting the recruitment of neutrophils to form collaborative clusters (111). The regulatory effects of flavonoids on intracellular and extracellular ROS show their divergent roles in different disease contexts.

### 3.3 Dendritic cells

Dendritic cells (DCs) are essential antigen-presenting cells that orchestrate immune responses by modulating T cell activation and differentiation, serving as a crucial connection between innate and adaptive immunity. Impairment of DCs has been demonstrated to play a role in the onset of inflammatory and autoimmune disorders. Recent studies indicate that flavonoids can exert immunomodulatory effects by further influencing T cells through the regulation of the immune response of DCs. Silibinin has been proven to inhibit the upregulation of co-stimulatory molecules and major histocompatibility complex (MHC) class II molecules on lipopolysaccharide (LPS) stimulated mature DCs, while also suppressing the release of pro-inflammatory cytokines IL-12, IL-23, and TNF-α, as well as CD4^+^ T cell proliferation and Th1/Th17 function, indicating strong immunosuppressive activity (112). RelB is a critical protein in the NF-κB pathway. Apigenin has been shown to inhibit RelB expression and nuclear translocation, affecting DC maturation and antigen presentation, which alters T cell responses from pro-inflammatory Th1/Th17 phenotypes to regulatory T cells (Tregs), thus alleviating inflammatory responses (113). Galangin and naringin have also been shown to contribute to this tolerance-inducing process. Treatment of DCs with galangin results in decreased expression of CD86 and MHC II molecules, with increased expression of programmed death ligand 1 (PD-L1) and IL-10 secretion. This promotes T helper (Th) cells differentiation into Tregs, reducing inflammatory responses (114). Conversely, naringin has been shown to promote the differentiation and maturation of DCs through the KBP4/NR3C1/NRF2 axis, thus providing a novel therapeutic approach for NRF2-dependent autoinflammatory conditions (115).

### 3.4 T cells

T cells are a key immune cell of the adaptive immune system with the capacity of controlling immunological responses. CD4^+^ T cells, also known as Th cells, can differentiate into Th1, Th2, Th17, T follicular helper cells (Tfh), and Tregs, regulating immunological responses throughout the body collaboratively (116). Our previous study demonstrated that phloretin inhibits glucose uptake and proliferation in activated CD4+ T cells and modulates the Th17/Treg balance through AMPK signaling pathway (117). Cytotoxic T lymphocytes (CTLs), also known as CD8^+^ T cells, are responsible for eliminating contaminated and cancer cells by releasing perforin and granzyme, leading to the initiation of apoptosis through interactions between Fas and Fas ligand (118). Dysfunction of T cell subsets and activities may cause the occurrence of autoimmune illnesses and tumor immune escape. Flavonoids can help restore immune homeostasis by regulating T cell differentiation. Apigenin is found to induce apoptosis in CD8^+^ T cells and decrease levels of inflammatory cytokines, including IL-6, IFN-γ, and IL-17, by inhibiting the STAT3/IL-17 signaling pathway. This effectively downregulates CTLs activity and alleviates tissue damage (119). Naringin shows promise in the treatment of autoimmune hepatitis by regulating essential genes associated with T cell responses, such as IL-6 and TNF (120).

T cell exhaustion represents a considerable challenge in cancer treatment. The expression of immune checkpoint molecules, PD-L1 and indoleamine 2, 3-dioxygenase1 (IDO1), is responsible for this process (121). Tumor cells frequently enhance the expression of these molecules to evade immune detection. Flavonoids have been shown to have the ability to recover T cell function by regulating the status of immune checkpoints. Myricetin can reduce the expression of PD-L1 and IDO1 induced by IFN-γ in lung cancer cells, by targeting the JAK-STAT-IRF1 signal pathway. This reduction can help recover T cell proliferation and effector activity (122). Icariin has been demonstrated to directly induce ferroptosis in colorectal cancer cells while simultaneously promoting CD8^+^ T cell activation and IFN-γ secretion, thereby augmenting the therapeutic efficacy of programmed cell death protein1 (PD-1) checkpoint inhibitors (123). Baicalin has been shown to improve the CD8^+^ T cell/Treg ratio against resistance to anti-PD-1 therapy via modifying the composition of the gut microbiota (124). Furthermore, flavonoids can augment the immune system’s capacity to combat tumors by modulating T cell autophagy. Baicalin has been shown to promote the degradation of CD274 within autolysosomes, a process facilitated by increased interaction between CD274 and LC3, which shows the potential of baicalin to interfere as a CD274 inhibitor to tumors (125).

### 3.5 B cells

B cells are also a key part of the adaptive immune system, producing antibodies that fight infections, however, disorders may lead to autoimmune diseases and allergy. Flavonoids have been shown to have the capacity to regulate the balance and function of B cells. Baicalin can lower the levels of pro-inflammatory cytokines (e.g., IL-1β, IL-2, IL-4, IL-6, IL-17A, and TNF-α) and chemokines, by regulating the ratio of different types of B cells (decreasing the ratio of B220^+^ lymphocytes and increasing the ratio of memory B cells) and inhibiting TGF-β1 signaling, associated with fibrosis and immune regulation. This indicates that it might help prevent the growth of abnormal B cells and diminish immune system activation (126). Formononetin has been shown to suppress IgE production by B cells and mast cell activation by downregulating key signaling pathways, the JAK/STAT and PI3K/Akt pathways, suggesting that flavonoids may have a broader immunomodulatory role in allergy and IgE-related diseases (127).

In addition, flavonoids can also regulate mast cells, eosinophils, and other immune cells, thereby alleviating allergic reactions and autoimmune diseases (128, 129). Flavonoids can regulate multiple signaling pathways, which gives them the potential to exert a wide range of effects on different immune cells. Their roles as antioxidants, which is another common mechanism they act on, can directly eliminate ROS from macrophages and neutrophils or stimulate the body’s intrinsic antioxidant responses to alleviate inflammation. Additionally, they can modulate macrophages via the Nrf2/HO-1 pathway, inhibiting the dissemination of inflammatory signals. A notable observation is their capacity to trigger a transition in immune cells, including macrophages, dendritic cells, and T cells, from a pro-inflammatory to an anti-inflammatory state during periods of inflammation, which occurs irrespective of the specific cell type. However, in the presence of tumors, flavonoids have been observed to induce a pro-inflammatory transformation in immune cells.

## 4 Flavonoids’ therapeutic applications in inflammatory diseases

Flavonoids have been demonstrated to exert regulatory effects on immune cells and inflammatory factors. Accumulating evidence from existing RCTs indicates that flavonoids may serve as adjunctive therapies for inflammatory diseases (**Table 2**). The following section provides a detailed overview of the therapeutic mechanisms of flavonoids in inflammatory diseases.

**Table 2:** RCTs of flavonoids in the treatment of inflammatory diseases.

Diseases	Flavonoids	Dose	Effect	Efficacy variables	Sample size and region	References
RA	Silymarin (50-80% Silybin)	300 mg/day	positive	The number of tender and swollen joints, duration of morning stiffness, severity of pain, disease activity, and disability indices, European League Against Rheumatism (EULAR) responses, levels of fatigue, depression, and anxiety↓	n=122 Romania	Zugravu GS et al. (130)
	Puerarin	400 mg/day	positive	Carotid intima-media thickness↓; The homeostasis model assessment of insulin resistance (HOMA-IR) ↓	n=119 China	Yang M et al. (131)
	Baicalin	500 mg/day	positive	Triglycerides, total cholesterol, LDL-cholesterol, apolipoproteins, CT-1 and hs-CRP ↓; Proportion of eligible patients maintaining good/moderate EULAR response after treatment ↑	n=351 China	Hang Y et al. (132)
	Quercetin	500 mg/day	positive	Early morning stiffness (EMS), morning, and after-activity pain ↓; DAS-28 and HAQ scores ↓; Plasma tumor necrosis factor-alpha (TNF-α) level ↓	n=50 women only Iran	Javadi F et al. (133)
	Quercetin + vitamin C	166 mg + 133 mg, tid	negative	Serum levels of TNF-α, interleukin-1beta (IL-1beta), interleukin -6(IL-6), C-reactive protein (CRP); Korean Health Assessment Questionnaire (KHAQ), Visual Analogue Scale(VAS).	n=20 (19 women) Korea	Bae SC et al. (134)
	Alpha-glucosylhesperidin (Hsp-G)	3 g/day	positive	ACR20 response rate ↑	n=19 Japan	Kometani T et al. (135)
Ulcerative Colitis (UC)	Anthocyanin-Rich Extract	800–1000 mg Anthocyanin, three times daily	negative	Clinical response rate, Mayo score	n=24 Swiss	Biedermann L et al. (136)
	Silymarin	140 mg/day	positive	Hemoglobin level ↑, erythrocyte sedimentation rate ↓; Disease activity index (DAI) ↓	n=70 Iran	Rastegarpanah M (137)
Irritable bowel syndrome	Soy isoflavones	40 mg/day	positive	TNF-α level, NF-κb level, fecal serine protease enzyme activity ↓	n=92 only women Iran	Jalili M et al. (138)
Obesity	Flavonoid-enriched juice	200 mL/day	positive	Weight, body mass index (BMI), fat mass, and waist circumference ↓; low-density lipoprotein cholesterol (LDL-C), glycated hemoglobin (A1c) levels, interferon gamma (IFNγ), TNF-α, leptin, plasminogen activator inhibitor (PAI-1) ↓; Glutathione peroxidase 1 (GPX1), adiponectin ↑	n=42 Spain	Navajas-Porras B et al. (139)
Non-alcoholic fatty liver disease (NAFLD)	Hesperidin	1 g/d	positive	Plasma levels of alanine aminotransferase, fasting blood glucose (FBG), fatty liver index (FLI), HOMO-IR, quantitative insulin sensitivity check index (QUICKI) ↓	n=92 Iran	Yari Z et al. (140)
Type 2 diabetes mellitus	Anthocyanin + dietary fibre	0.28 g/day + 1.26 g/day	positive	Fasting blood glucose (FBG), glycated haemoglobin (HbA1c), low density lipoprotein (LDL) ↓; glomerular filtration rate ↑	n=60 Thailand	Teparak C (141)
	Rutin	500 mg/day	positive	FBG, insulin, HbA1c, homeostasis model assessment of insulin resistance (HOMO-IR), low-density lipoprotein cholesterol (LDL-c), total cholesterol (CHOL), LDL.HDL ratio, atherogenic index of plasma (AIP), malondialdehyde (MDA), IL-6 ↓; quantitative insulin sensitivity check index (QUICKI), HDL-c, total antioxidant capacity (TAC) ↑	n=50 Iran	Bazyar H (142)
Hyperglycemia	Eriomin (a mix of eriocitrin plus other citrus flavonoids)	200 mg/day	positive	Blood glucose, HOMO-IR, glucagon, interleukin-6, TNF-α, and alkaline phosphatase ↓; Glucagon-like peptide 1 (GLP-1), triglycerides ↑	n=45 Brazil	Cesar TB et al. (143)
Multiple sclerosis (MS)	EGCG + coconut oil	800 mg/day + 60 ml/day	positive	IL-6, fat percentage ↓; Butyrylcholinesterase enzyme (BuChE), β-hydroxybutyrate (βHB), Paraoxonase 1 (PON1), albumin and functional capacity (the Expanded Disability Status Scale (EDSS)) ↑	n=51 Spain	de la Rubia Ortí JE et al. (144)
Bronchial pneumonia	Naringenin	5 mg/kg, 5 days	positive	Symptom disappearance time, the incidence rates of complications, the incidence rates of adverse events and the level of IL-6, IL-8, TNF-α↓; IL-10↑	n=180 China	Yao W et al. (145)
COVID-19	Nano-silymarin	210 mg/day	negative	Symptoms resolution time, laboratory parameters, and hospitalization duration	n=50 Iran	Aryan H (146)
Chronic pancreatitis	Soy bread (isoflavones)	3 slices/day (99 mg/d)	positive	TNF-α↓	n=10 USA	Ahn-Jarvis J et al. (147)
Alzheimer’s disease (AD)	Silymarin	450 mg/day	positive	Catalase and malondialdehyde serum levels ↓, Mini Mental State Exam (MMSE) ↑	n=33 Iran	Navabi SM et al. (148)
Traumatic brain injury	Enzogenol	1000 mg/day	positive	Incidence of cognitive failure (mean CFQ score) after 6 weeks ↓	n=60 New Zealand	Theadom A (149)
Asthma	Soy isoflavones	98 mg/day	positive	The number of severe asthma exacerbations in asthmatic patients with the high PAI-1-producing genotype ↓	n=265 USA	Cho SH et al. (150)
Atopic dermatitis	Licochalcone A lotion	0.025%	positive	The relapse rate ↓	n=26 Thailand	Udompataikul M et al. (151)
Hypertension	hesperidin-enriched orange juice	600 mg/day	positive	Systolic blood pressure, pulse pressure↓	n=159 Spain	Valls RM et al. (152)

### 4.1 Anti-inflammatory effects in inflammatory bowel disease

IBD is a chronic intestinal disorder closely linked to immune system dysfunction. Corticosteroids and 5-aminosalicylic acid are conventional IBD medications, but they sometimes have limited effectiveness and may cause side effects such as diabetes and kidney problems (153). Recent immunosuppressants and biologics have demonstrated efficacy for numerous patients; however, they also elevate the risk of severe cardiovascular complications, including thrombosis (154). Flavonoids are a relatively harmless type of medicine that may help treat IBD. Cohort studies have found a correlation between high intake of anthocyanins and quercetin and a lower risk of ulcerative colitis (UC) (155, 156). In patients with moderate to severe UC, treatment with anthocyanin-rich extracts has been shown to lower calprotectin levels, indicating therapeutic benefits. However, these extracts were less effective in patients with Crohn’s disease (136). Although studies have shown that anthocyanins do not have a significant benefit on Crohn’s disease, anthocyanin-rich maqui extract inhibited the NLRP3 inflammasome and mast cell activation in mice with TNBS-induced colitis (157). Therefore, further research is needed to understand the role of anthocyanins in Crohn’s disease. Flavonoids have been shown to have positive effects on colitis by modulating immune cell activity and preventing them from becoming pro-inflammatory. In a DSS mouse model of colonic lesions, puerarin has been found to directly inhibit M1 polarization of macrophages, while luteolin and formononetin have been demonstrated to regulate the M1/M2 balance of macrophages (87, 158, 159). Some flavonoids below have been reported to alleviate colitis through various mechanisms. EGCG can inhibit Th1 cell polarization, while hyperoside can regulate the Th17/Treg balance, hesperidin helping rebalancing Th1/Th2 and Th17/Treg, and wogonin has been found to activate the AhR pathway, thereby preventing ILC3 from transforming into ILC1 (160–163). Flavonoids may also regulate the immune system and help prevent and treat inflammatory bowel disease by interacting with gut microbiota in bidirectional interactions. On one hand, following colon entry, flavonoid metabolites display gut microbiota-dependent characteristics, such as equol, a metabolite derived from soy isoflavones, which forms entirely dependent on the presence and composition of gut microbiota (164, 165). On the other hand, flavonoids entering the intestine also affect the composition of the gut microbiota (166). This effect not only directly influences the abundance of IBD-related gut microbiota but also helps gut microbiota to produce short-chain fatty acids (SCFAs), regulators of T lymphocytes, macrophages, and neutrophils, contributing to gut homeostasis (167–170). Taxifolin has been demonstrated to modify gut microbiota composition, which can lead to increased SCFAs production, thereby suppressing the production of TNF-α, IL-1β, and IL-6 in colonic tissue, thus ameliorating DSS-induced colitis (171).

Flavonoids may also play a role in preventing the progression of UC to colorectal cancer. Glabridin has been demonstrated to inhibit STAT3 phosphorylation, regulate MMP1/3 activity, alleviate inflammation, reduce extracellular matrix degradation and decrease the occurrence of epithelial-mesenchymal transitions (172). Neohesperidin (NHP) can inhibit macrophage migration into target tissues and decrease pro-inflammatory cytokine levels, including TNF-α, IL-1β, IL-6, and COX-2, at both mRNA and protein levels, exceeding that of mesalazine at similar concentrations (173). Vitexin has been shown to reduce the quantity of M1 phenotype macrophages in healthy tissue adjacent to tumors, enhance M1 macrophage polarization within tumor tissue, and impede the progression from ulcerative colitis to colorectal cancer (174).

### 4.2 Anti-inflammatory effects in rheumatoid arthritis

RA is a chronic inflammatory disease that causes persistent inflammation of the synovial membrane and joint damage, potentially resulting in disability (175). Although the use of traditional medications, DMARDs, and innovative biologic agents has collectively improved treatment outcomes, numerous patients exhibit insufficient responses to existing therapies or adverse side effects, highlighting the necessity for novel treatment strategies (176). Recent evidence from epidemiological and clinical studies suggests that dietary flavonoids may play an important role in RA treatment. A study from the US National Health and Nutrition Examination Survey (NHANES) indicated that adults in the US with elevated flavonoid consumption may exhibit a reduced likelihood of developing RA (177). Furthermore, individuals with active rheumatoid arthritis may experience alleviation of their condition and a reduction in inflammatory markers due to specific flavonoid compounds or flavonoid-rich extracts, such as silymarin and curcumin, with effects superior to those of DMARDs (130, 178).

Flavonoids may assist in the treatment of RA by regulating immune dysregulation in the synovial microenvironment. A key factor in RA development is the imbalance between pro-inflammatory effector T cells and anti-inflammatory Tregs, where flavonoids can directly correct and restore balance in the immune system. Naringin can inhibit the migration and polarization of CD4^+^ T lymphocytes in the synovium by regulating mitochondrial fission (179). In a collagen-induced arthritis (CIA) model, kaempferol was found to reduce the quantity of pathogenic CD4+ effector memory T cells while increasing naïve T cells and Tregs, thereby ameliorating the condition (180), while berberine and rutin augment Treg activity to assist in the management of RA (181). Kurarinone inhibits the differentiation of pro-inflammatory Th1 and Th17 cells by directly activating the antioxidant Nrf2/KEAP-1 pathway, thus alleviating RA symptoms (182). Morin alleviates RA by inhibiting Th17 differentiation and fatty acid synthesis following PPARγ activation (183). Hesperidin alleviates RA by inhibiting Th17 activation, as well as reducing serum levels of TNF−α, IL−6, and IL−17A, ultimately improving joint pathology and clinical score (184). In the adjuvant-induced arthritis (AIA) rat model, Cyanidin enhances the outcomes of RA by reestablishing the Th17/Treg balance and suppressing the differentiation of Tfh cells via ROCK2 signaling (185). This targeted regulation of T cell subsets is crucial for attaining sustained remission of RA and is closely related to the control of autoreactive T cells (186). Macrophages also play an important role in coordinating RA synovitis and joint degradation (187). Flavonoids can prevent pro-inflammatory macrophages from functioning and promote their conversion into anti-inflammatory cells, thereby combating arthritis. Hesperidin inhibits macrophage infiltration into tissues by diminishing COX-2 expression (188). Acacetin binds specifically to the ATP domain of HSP90, causing HSP90 to detach from COX-2, which leads to COX-2 being ubiquitinated and broken down in macrophages, thereby reducing inflammatory reactions (189). Icariin, delivered via exosomes from mesenchymal stem cells, has been shown to convert pro-inflammatory M1 macrophages in synovial tissue into anti-inflammatory M2 macrophages, at the same time inhibiting glycolysis (190). Flavonoids can also prevent abnormal crosstalk between different immune cell groups that triggers the inflammatory cascade in RA (191). An adverse feedback loop exists between T cells and macrophages, wherein cytokines produced by T cells, such as IL-17A, diminish the function of synovial macrophages and worsen inflammation. Cyanidin has been shown to suppress this IL-17A-mediated impairment, thereby halting this intercellular inflammatory transmission (192). Neutrophils are essential in the pathogenesis of RA and represent an important target for therapeutic intervention, as they perpetuate inflammation through the secretion of cytokines, chemokines, and reactive oxygen species, in addition to forming NETs (193). Flavonoids can prevent these harmful processes to treat RA. Quercetin can inhibit neutrophil infiltration into tissues, induce apoptosis in active neutrophils, and obstruct NETs formation by interfering with autophagy (194). Rutin can obstruct neutrophil migration to joints and suppress the local synthesis of TNF-α (195). Refractory RA is when an individual with RA fails to respond to DMARDs treatment (196). The overexpression of the drug efflux pump P-glycoprotein (P-gp) in lymphocytes is regarded as a mechanism of DMARDs resistance (197). Nobiletin, functioning as a P-gp inhibitor, can suppress P-gp overexpression in lymphocytes in the AIA rat model when co-administered through a nanodelivery system, providing novel insights for refractory RA (26, 198). Flavonoid compounds may also exert beneficial effects on the overall integrity of joint structures. For instance, they can promote chondrocyte differentiation via the MAPK signalling pathway, inhibit synovial autophagy and fibroblast-like synoviocyte activation, making flavonoids even better candidates for comprehensive anti-arthritic agents (199–201).

### 4.3 Anti-inflammatory effects in metabolic disorders

Metabolic disorders, such as obesity, type 2 diabetes (T2DM), and atherosclerosis, are caused by a persistent low-grade inflammatory condition known as ‘metabolic inflammation’ (202). Metabolic stress activates immune cells, such as macrophages and neutrophils, in metabolic organs, including adipose tissue, the liver, the pancreas, and blood vessels (203). These activated immune cells produce cytokines, contribute to cellular damage, and promote insulin resistance. Analysis of plasma samples from T2DM patients adhering to a Mediterranean diet for 12 weeks revealed an elevation in plasma concentrations of naringin, hesperetin and hesperidin, resulting in a reduction of inflammation and oxidative stress in individuals with T2DM (204). Flavonoids can regulate immune cells to inhibit the progression of metabolic inflammation.

#### 4.3.1 Anti-inflammatory effects in obesity and fatty liver

Adipose tissue serves as the primary site of inflammation in metabolic inflammatory disorders. Flavonoids can reduce inflammation by regulating macrophage polarization, as the infiltration of macrophages and pro-inflammatory (M1) polarization are hallmark traits of obesity (205). In obese mice, dietary supplementation with kaempferol has demonstrated a reduction in intestinal inflammation and a decrease in the influx of immune cells into the intestines, thereby mitigating systemic metabolic issues (206). Apigenin, when delivered in nanoparticle delivery systems, promotes the transition of adipose tissue towards the M2 phenotype, thereby reducing obesity-related inflammation (207). 7, 8-Dihydroxyflavone and baicalin are two flavonoids with similar effects, blocking key inflammatory signaling pathways, including NF-κB and JNK, in macrophages, reducing insulin resistance in overweight individuals (208, 209). When metabolic inflammation extends to the liver, it may result in non-alcoholic fatty liver disease (NAFLD), a condition for which flavonoids demonstrate significant protective potential. Nobiletin, a citrus flavonoid, inhibits hepatic lipid accumulation and decelerates the progression of NAFLD by regulating M1/M2 macrophage polarization through enhanced autophagy and by suppressing the NLRP3 inflammasome, a critical contributor to inflammatory cell death (210). Hyperoside similarly promotes M2 macrophage polarization in an Nr4A1-dependent manner, contributing to the amelioration of NAFLD (211). Researchers have discovered that green tea extract, Theaphenon E (TE), protects the liver by diminishing lipid accumulation and preserving elevated CD4^+^ T cell counts (212).

#### 4.3.2 Anti-inflammatory effects in diabetic vascular complications

Flavonoids can help prevent inflammation that may potentially harm the pancreatic beta cells. A study found that a flavonoid-rich diet, mixing cocoa powder and carob flour, may preserve β-cell mass in diabetic rats through preventing macrophages from infiltrating into the pancreatic islets and inhibiting NF-κB-mediated inflammation. This approach may help regulate insulin secretion and delay the development of T2DM (213). The persistent inflammation and hyperglycemia associated with diabetes inevitably have severe effects on blood vessels and tissues, leading to a series of serious complications (214). Flavonoids can alleviate these complications by regulating immune cell-mediated immune responses in affected tissues. Kaempferol mitigates diabetic retinopathy by targeting retinal microglia, which are the resident macrophages of the central nervous system. Kaempferol significantly diminishes the pro-inflammatory response of microglia and promotes an anti-inflammatory M2-like phenotype, thereby contributing to maintaining retinal integrity (215). Hyperoside may help treat diabetic nephropathy by regulating the T cell balance towards anti-inflammatory Th2 and Treg populations and by promoting macrophage polarization from the M1 to the M2 phenotype (216). Cardamonin inhibits diabetic cardiomyopathy by preventing M1 macrophages from infiltrating the cardiomyocyte and modifying their morphology. Meanwhile, cardamonin can activate KEAP1 to release the transcription factor NRF2, which activates a strong protective program that shields cardiomyocytes from damage by weakening inflammation and free radicals (217).

#### 4.3.3 Anti-inflammatory effects in cardiovascular diseases

Inflammatory responses are key drivers of the development and progression of cardiovascular diseases. Immune cells contribute to pathological cardiovascular remodeling by inducing endothelial dysfunction, vascular remodeling, cardiomyocyte injury, and fibrotic changes (218, 219). Moreover, immunosenescence has emerged as an important contributor to the pathogenesis of atherosclerosis and hypertension (220). Flavonoids exert multiple protective effects in cardiovascular inflammatory conditions. In the vascular endothelium, flavonoids promote endothelial cell survival and function (221, 222). As for cardiomyocytes, they regulate autophagy and alleviate cellular swelling and fibrosis in heart failure conditions (222–224). Within vascular smooth muscle cells, flavonoids suppress their proliferation, migration, and inflammatory activation (225). Furthermore, by regulating the gut microbiota, flavonoids strengthen intestinal barrier integrity and thereby mitigate microbiota-driven cardiovascular pathology (226). Beyond their suppression of general inflammatory mediators, flavonoids can specifically target disease-relevant inflammatory markers, highlighting their therapeutic potential in managing cardiovascular inflammation (227).

Atherosclerosis is an inflammatory arterial disorder that may lead to strokes and myocardial infarctions. Flavonoids can not only inhibit cytokines but also regulate macrophage metabolic reprogramming, thereby preventing the formation of lipid-laden ‘foam cells’ that contribute to the development of atherosclerotic plaques (228). Wogonin, a flavonoid isolated from Scutellaria baicalensis, activates the PPARα-KLF11-YAP1 transcription complex, which redirects macrophage energy metabolism away from pro-inflammatory glycolysis towards protective fatty acid oxidation, thereby inhibiting foam cell formation and alleviating plaque inflammation (229). Quercetin can limit pyroptosis of macrophages by blocking the KEAP1/NRF2 interaction (230). Hawthorn leaf flavonoids can inhibit sPLA2-IIA in macrophages, thereby reducing macrophage inflammation (231). Both mechanisms have been shown to slow atherosclerosis progression in ApoE−/− mice fed a high-fat diet by targeting macrophages. In terms of reducing the formation of atherosclerotic plaques, biochanin A (BCA) promotes macrophage cholesterol efflux, while kaempferol suppresses macrophage inflammatory responses (232, 233). Elevated blood glucose levels prompt neutrophils to release NETs, resulting in vascular damage. Prenylchalcones may inhibit this process, indicating their potential as supplementary therapies for metabolic disorders (234).

Hypertension is also strongly associated with immune activation and chronic inflammation. Aberrant immune responses promote vascular inflammation, leading to endothelial dysfunction, vascular remodeling, and increased peripheral vascular resistance, which eventually drive blood pressure elevation (235, 236). Cohort studies indicate that flavonoids not only reduce the risk of developing hypertension but also prevent hypertension-related target organ damage and improve long-term clinical outcomes (237–240). The paraventricular nucleus (PVN) of the hypothalamus, a bilateral structure adjacent to the third ventricle, plays a critical role in central blood pressure regulation. Beyond their anti-inflammatory effects, flavonoids can regulate immune and inflammatory processes within the PVN through actions on immune cells, thereby contributing to blood pressure control. Puerarin and anthocyanins attenuate blood pressure elevation in salt-induced prehypertensive rats by suppressing NLRP3 inflammasome activation and ROS production in the PVN (241, 242). Similarly, luteolin ameliorates hypertension by inhibiting NF-κB-mediated inflammatory signaling and the PI3K/Akt pathway within the hypothalamic PVN (243).

### 4.4 Anti-inflammatory effects in neuroinflammation and neurodegenerative diseases

Neuroinflammation is characterized by immune cells in the central nervous system (CNS), mainly microglia and astrocytes, remaining persistently activated, which is regarded as a predominant mechanism in numerous neurodegenerative and neurological diseases (244). Microglia can exert neuroprotective effects by phagocytosing harmful protein aggregates; however, excessive phagocytosis of these aggregates may lead to neuronal damage and disease progression (245). In pathological conditions, peripheral immune cells, such as T cells and neutrophils, may also infiltrate the brain and spinal cord, further intensifying the inflammation (246, 247). Flavonoids can alleviate neuroinflammatory processes and decrease the progression of neurodegenerative diseases through their regulatory effects on immune cells, particularly microglia (248).

#### 4.4.1 Anti-inflammatory effects in Alzheimer’s disease

In Alzheimer’s disease (AD), amyloid-β (Aβ) plaques and tau protein tangles chronically activate microglia, causing a neurotoxic inflammatory state and impairing their phagocytic function (249). A study based on the NHANES database found that adults in the US population with higher flavonoid consumption exhibited a lower risk of AD-related mortality (250). Myricetin may aid in treating AD by blocking the p38 MAPK pathway and activating the NLRP3 inflammasome in microglia, thereby reducing Aβ accumulation (251). In addition to suppressing pathological processes, flavonoids can also enhance microglia’s protective ability. Cyanidin-3-O-Glucoside (C3G) facilitates the transition of microglia from the M1 to the M2 phenotype by activating the PPARγ signal pathway and enhancing the phagocytosis of Aβ42 by upregulating TREM2, thereby facilitating the clearance of accumulated β-amyloid (252). Rutin has also been shown to help microglia uptake extracellular tau oligomers, thereby directly combating AD (253). Microglial senescence, a functional disorder state associated with aging and AD, can be mitigated through preventive measures, thereby alleviating neuroinflammation and cognitive decline (254). Studies suggest that delphinidin may decelerate this process through the AMPK/SIRT1 pathway, potentially mitigating cognitive deficits and pathological features of AD (255).

#### 4.4.2 Anti-inflammatory effects in demyelinating diseases

Multiple sclerosis (MS) is an autoimmune demyelinating disease in which both T cells and glial cells contribute to the progression of the disease (256, 257). Flavonoids have shown their potential for therapeutic applications in MS via regulating both the central and peripheral immune systems. In the experimental autoimmune encephalomyelitis (EAE) model of MS, procyanidin B2 3, 3’’-di-O-gallate (PCB2DG) has been found to directly suppress the immune response of pathogenic CD4^+^ T cells, aiding in disease management. Glycolysis is crucial for T cell activation. PCB2DG exhibits anti-inflammatory effects by modulating glycolytic metabolism, consequently reducing the production of T cell-mediated inflammatory cytokines such as IFN-γ and IL-17, as well as their infiltration into the spinal cord. This mechanism has been proved effective for treating spinal cord injury (258). Baicalein and kaempferol have been demonstrated to alleviate demyelination and microglial activation caused by cuprizone poisoning by inhibiting the STAT3 and NF-κB signaling pathways (259). Agathisflavone has been shown to influence microglia activation through the estrogen receptor alpha, promoting myelin regeneration and alleviating symptoms of MS (260).

#### 4.4.3 Anti-inflammatory effects in acute central nervous system injury

After acute injuries such as stroke and trauma, the activation of microglia and the infiltration of peripheral immune cells into ischemic tissues may trigger a robust and deleterious neuroinflammatory response (261). Flavonoids have been proven to have therapeutic potential in repairing such damage. Quercetin can regulate the PI3K/Akt/NF-κB signaling pathway, promoting a transition from a pro-inflammatory M1 phenotype to an anti-inflammatory M2 phenotype in microglia and macrophages, thereby effectively alleviating brain ischemia/reperfusion injury (85). In spinal cord injury models, alpinetin reduce neuroinflammation and enhance motor function recovery by inhibiting microglial activation (262). In an experimental intracerebral hemorrhage model, didymin has been found to improve cerebral function by reducing microglial activation and pyroptosis. Furthermore, this process also leads to a reduction of neutrophils infiltrating the perihematomal tissue, thereby helping to alleviate post-hemorrhagic neuroinflammation (263).

#### 4.4.4 Anti-inflammatory effects in mental, stress and pain-related disorders

Neuroinflammation is considered a pathogenic factor in the development of depression and anxiety (264), while flavonoids have been demonstrated to exert therapeutic effects on depression and anxiety by reducing neuroinflammation induced by microglia. Luteolin can promote the Arg-1+ microglial phenotype in a chronic stress-induced depression model, which contributes to the suppression of microglial pro-inflammatory activity and ameliorates depressive-like behavior (265). Hyperoside has been demonstrated to ameliorate depressive-like behavior by promoting M2 polarization of microglia in the hippocampus (266), and quercetin can alleviate cognitive impairments in depressed mice by targeting HSP90 to inhibit the activation of the NLRP3 inflammasome in microglia (267). In a mouse model of chronic unpredictable stress (CUS) depression, morin can relieve neuropathic pain by balancing M1/M2 microglial polarization and inhibiting neuroinflammation (268).

A major impediment in the treatment of neurological disorders is the blood-brain barrier, while delivery mechanisms can enhance the efficacy of flavonoids. Xu et al. formulated BDNF(brain-derived neurotrophic factor)-quercetin alginate nanogels and delivered them intranasally, resulting in a nearly 50-fold enhancement in the bioavailability of quercetin compared to oral treatment (269). Zhang et al. attached the blood-brain barrier-penetrating peptide (RVG29) to the surface of nanoparticles and included 4, 4’-dimethoxychalcone (DMC), referred to as RVG-nDMC. This delivery system successfully traversed the blood-brain barrier (BBB), improved the transport of DMC to dopaminergic neurons and microglia in the substantia nigra pars compacta of Parkinson’s disease (PD) mice, and intervened in PD (270).

### 4.5 Anti-inflammatory effects in respiratory tract diseases

When pathogens invade the respiratory tract, innate immune cells respond to the infection rapidly. However, excessive activation might result in inflammation and damage to the respiratory system (271). Consequently, in respiratory tract infections, it is essential for the immune system to maintain a balance between combating pathogens and minimizing host tissue damage (272). Flavonoids have shown potential in this condition by directly reducing pathogen replication and virulence while also regulating the host immune response, offering new prospects for the treatment of respiratory tract infections (RTI).

#### 4.5.1 Anti-inflammatory effects in acute lung injury

Acute lung injury (ALI) and its more severe form, acute respiratory distress syndrome (ARDS), are serious conditions usually caused by sepsis, trauma, or severe infection. ALI is characterized by widespread pulmonary inflammation, alveolar edema, and progressive respiratory failure, with neutrophils and macrophages serving as key cellular mediators in this process (273, 274). Numerous flavonoids have demonstrated considerable therapeutic potential in experimental models of ALI and sepsis, particularly in sepsis-induced acute lung injury. In ALI, macrophages typically exhibit a pro-inflammatory M1 phenotype, secreting substantial quantities of cytokines and aggravating lung tissue damage. Flavonoids may counteract this pathological process by promoting the transition of macrophages toward the anti-inflammatory M2 phenotype, thereby facilitating inflammation resolution and initiating tissue repair mechanisms. Wogonin and apigenin have been reported to ameliorate sepsis-induced ALI by regulating macrophage M1/M2 polarization (275, 276). Oroxylin A alleviates sepsis through the induction of “trained immunity”. It increases LC3-associated phagocytosis (LAP) in macrophages via activation of the Dectin-1-Syk signaling axis and the mTOR pathway, thereby enhancing macrophage capacity to combat infection and resist sepsis progression (277). Catechin hydrate directly inhibits macrophage RasGRP1, a pro-inflammatory gene in macrophages, thereby diminishing excessive inflammation and oxidative stress that contribute to numerous organ dysfunctions resulting from sepsis (278). Icariin II mitigates LPS-induced ALI by targeting the neutrophil receptor CXCR, hence reducing excessive neutrophil activation and the formation of NETs (279).

#### 4.5.2 Anti-inflammatory effects in bacterial respiratory tract infections

With the continuous advancement of antibiotic therapies, bacterial pneumonia has been partially controlled. However, the emergence of drug resistance poses significant challenges to both clinical treatment and public health, particularly in the context of the COVID-19 pandemic, during which the prevalence of multidrug-resistant organisms (MDROs) has increased rapidly (280, 281). Flavonoids provide a multi-target therapeutic strategy for bacterial pneumonia through various mechanisms, including direct antibacterial effects, suppression of bacterial pathogenicity, and regulation of the host’s immunological response to infection. Pseudomonas aeruginosa is an opportunistic Gram-negative bacterium frequently responsible for hospital-acquired pneumonia and may lead to severe and often multidrug-resistant infections (282). In a murine model of bacterial pneumonia, luteolin demonstrated the capacity to inhibit M1 macrophage polarization by suppressing the EGFR/PI3K/AKT/NF-κB and EGFR/ERK/AP-1 signaling pathways. It can simultaneously reduce lung permeability, neutrophil infiltration, pro-inflammatory cytokine synthesis, and pulmonary bacterial load, thereby improving the survival rate of mice (283). Phloretin exhibits direct antibacterial and anti-biofilm properties against non-typeable Haemophilus influenzae (NTHi), Moraxella catarrhalis, and Streptococcus pneumoniae. In murine models, dietary supplementation with phloretin was found to decrease NTHi bacterial load and reduce levels of the neutrophil chemoattractant CXCL1, thereby ameliorating pneumonia (284). MgrA is a key regulatory factor in Staphylococcus aureus and plays a central role in regulating bacterial virulence and resistance. methylophiopogonanone can diminish the connection between MgrA and DNA in S. aureus, leading to reduced toxin expression, diminished bacterial adhesion, disruption of immune evasion mechanisms, and enhanced neutrophil chemotaxis, thereby preventing S. aureus-induced pneumonia in mice (285).

In addition to their therapeutic effects on existing infections, flavonoids also demonstrate preventive potential against bacterial infections. EGCG elicits an adjustable pro-inflammatory response in macrophages via the 67LR/p38/JNK signaling pathway, showing promise in the prevention of bacterial infections. Furthermore, it can also regulate macrophage-mediated immunity, stimulate phagocyte migration, and enhance the immune system’s capacity to respond more swiftly and effectively to subsequent bacterial assaults (100).

#### 4.5.3 Anti-inflammatory effects in viral respiratory tract infections

Whether it’s seasonal influenza or the more recent COVID-19 pandemic, various viral respiratory infections can elicit immune responses, resulting in pulmonary damage. Flavonoids present a multi-targeted therapeutic approach that not only inhibits viral invasion and replication but also regulates the host’s inflammatory response, thereby offering therapeutic benefits (286, 287). In an experimental model of lung inflammation induced by the H1N1 subtype of influenza A virus, an RNA virus responsible for seasonal and pandemic influenza, 5-methoxyflavone was shown to suppress the recruitment of CD8^+^ T cells, attenuate inflammatory responses, and reduce ALI (288). In a mouse model of H9N2 viral infection, baicalin was found to directly inhibit viral replication, augment the expression of antiviral proteins Mx1 and PKR, equilibrate the CD4^+^/CD8^+^ T cell ratio, ultimately strengthening mucosal immunity against H9N2. Although the H9N2 avian influenza virus exhibits low pathogenicity in humans compared to other avian influenza subtypes, these findings suggest potential implications for the prevention of highly pathogenic avian influenza (289). Luteolin-7-O-glucoside (LUT-7G) has been demonstrated to ameliorate lung injury and inhibit respiratory syncytial virus (RSV) replication by preserving mitochondrial function in alveolar macrophages and promoting the production of protective interferon-β (290).

The presence of COVID-19 not only leads to severe pneumonia and associated sequelae but also significantly affects the epidemiology and antimicrobial resistance patterns of other respiratory pathogens (291, 292). The cytokine storm in COVID-19 is an exaggerated immune response characterized by the excessive synthesis of pro-inflammatory cytokines, potentially resulting in ARDS and multi-organ failure. It is closely associated with the rapid deterioration of COVID-19 and high mortality rates (293). An RCT has shown that a flavonoid-containing mouthwash can effectively reduce the viral load of SARS-CoV-2 in saliva (294). Lianhua Qingwen Capsules, which have been clinically validated for the treatment of COVID-19, contain forsythoside as a main active ingredient, which includes rutin, quercetin, and other flavonoids, confirming the therapeutic efficacy of flavonoids against COVID-19 infection indirectly (295). Diosmetin-7-O-β-D-pyran glucoside (DG) and kaempferol can modulate M1/M2 macrophage polarization, suppress endotoxin-induced cytokine storms, enhance mouse survival rates, and concurrently limit viral activity and replication (296, 297). Furthermore, the combination of quercetin and dasatinib has been found to reduce the infiltration of macrophages and neutrophils in pulmonary tissue, thereby mitigating the inflammatory response in the lungs of patients with COVID-19 (298). It is worth noting that hesperidin and its aglycone metabolite hesperetin demonstrated antiviral effects at multiple stages of COVID-19 infection. Hesperetin can inhibit SARS-CoV-2 Spike -induced activation of the NLRP3 inflammasome, thereby attenuating excessive inflammatory responses (299). Hesperidin interferes with the binding between the spike protein and human angiotensin-converting enzyme 2 (hACE2), thus suppressing intercellular transmission and immune evasion of SARS-CoV-2 (300). Furthermore, hesperetin can directly inhibit SARS-CoV-2 replication (301). These findings indicate that hesperidin and hesperetin exert inhibitory effects across multiple phases of the SARS-CoV-2 life cycle. Moreover, hesperetin and hesperidin are reported to directly suppress viral replication in other pathogens such as RSV, zika virus (ZIKV), chikungunya virus (CHIKV), suggesting them as potential broad-spectrum antiviral agents with multi-target mechanisms (302, 303). These evidences highlight the therapeutic potential of hesperidin and hesperetin in managing viral infections and co-infections. Further investigation is warranted to elucidate the precise mechanisms by which hesperidin and hesperetin act at different stages of these viral infections.

Another important finding is that many flavonoids have been proven to significantly inhibit the release of elastase from neutrophils. Neutrophil elastase is a potent proteolytic enzyme secreted by neutrophils as part of the innate immune response to infection. However, overactivation can lead to structural damage to alveolar and vascular tissues, contributing to the pathogenesis of COVID-19 and other pulmonary disorders. The inhibition of elastase allows flavonoids to assume a protective function, improving clinical outcomes in multiple respiratory diseases (304).

### 4.6 Anti-inflammatory effects in asthma and allergic diseases

Asthma and allergic diseases are characterized by chronic inflammation and atypical immune responses, mediated through complex interactions among multiple immune cells, with both innate and adaptive immunity being essential. Asthma frequently co-occurs with other allergic diseases—such as allergic rhinitis, atopic dermatitis, and food allergies—posing significant challenges to accurate diagnosis and effective management, ultimately influencing patients’ quality of life (305). An RCT indicated that oral quercetin supplementation can effectively mitigate symptoms associated with allergic rhinitis induced by pollen (306). Furthermore, epidemiological evidence from a cohort study indicated an inverse association between maternal dietary intake of flavonoids during pregnancy and the subsequent risk of childhood asthma development (307). Flavonoids can markedly inhibit the release of histamine and pro-inflammatory cytokines from mast cells, which are central mediators in allergic responses (308). Through their immunoregulatory properties, flavonoids contribute significantly to the alleviation and management of various asthma and allergic diseases.

#### 4.6.1 Anti-inflammatory effects in asthma and allergic airway inflammation

Asthma is a chronic respiratory disorder, and recent years have witnessed significant progress in understanding the role of type 2 immunity in its pathogenesis. Type 2 immunity is an immune response driven by Th2 cells, resulting in the synthesis of allergen-specific immunoglobulin E (IgE) and the activation of mast cells and basophils (309). Flavonoids exhibit diverse therapeutic potential in targeting airway hyperresponsiveness, inflammation, and tissue remodeling in asthma. They can modulate β-catenin to inhibit epithelial-mesenchymal transition (EMT) and thereby mitigate airway remodeling in asthma (310). Additionally, they are capable of regulating the Th1/Th2 immune balance. Research has found that tectorigenin can reestablish this equilibrium in asthmatic mice by upregulating the expression of Th1-associated factors while suppressing Th2 cytokines such as IL-4, IL-5, and IL-13, consequently reducing serum IgE levels. This immunoregulatory effect has been linked to the activation of the antioxidant Keap1/Nrf2/HO-1 signaling pathway (311). Tectochrysin and sophoraflavanone G have also been found to suppress Th2 responses and augment the antioxidant capacity of lung tissue, thus ameliorating allergic airway inflammation (312, 313). Flavonoids additionally regulate key intracellular signaling pathways in both immune and structural cells. Daphnetin exerts anti-inflammatory effects by inhibiting intracellular Ca²^+^ mobilization and suppressing the JAK/STAT6 signaling pathway—a critical axis involved in Th2 cytokine production—thereby alleviating allergic airway inflammation (314). Wogonoside can significantly reduce airway inflammation and excessive mucus production while enhancing airway remodeling by inhibiting the activation of NF-κB and STAT6 in lung tissue and bronchial epithelial cells, as well as decreasing Th2-related cytokines (IL-4, IL-5, IL-13) in lung tissue and serum IgE levels (315). Moreover, flavonoids can regulate immune cells implicated in asthma pathogenesis. Tilianin suppresses Th2 immune responses by down-regulating interferon regulatory factor 4 (IRF4) in DCs, therefore mitigating house dust mite-induced allergic asthma (316).

#### 4.6.2 Anti-inflammatory effects in atopic dermatitis

Atopic dermatitis is a chronic and recurrent inflammatory dermatosis that belongs to the spectrum of atopic diseases, which also includes asthma and food allergies. In atopic dermatitis, an exaggerated type 2 immune response, predominantly facilitated by Th2 cells, can induce skin inflammation and provoke neural sensitization, resulting in intense pruritus (317). Flavonoids have demonstrated efficacy in alleviating symptoms by regulating skin-infiltrating immune cells and restoring epidermal barrier integrity. Research on a ternary compound formula containing ginsenoside Rg1, tetrandrine, and icaritin (GTI) can mitigate atopic dermatitis-like symptoms by reducing the infiltration of eosinophils, mast cells, and CD4^+^ T cells in skin tissue, as well as reducing IgE-mediated reactions and inhibiting MAPK signaling activation. Furthermore, it enhances epidermal barrier function by promoting the expression of tight junction proteins (318). Emerging technologies are being developed to overcome the challenge of flavonoid delivery across the skin barrier. A microneedle-based delivery system incorporating epigallocatechin gallate and L-ascorbic acid-loaded poly-γ-glutamic acid has been found to substantially ameliorate atopic dermatitis symptoms in mice, significantly decrease serum IgE and histamine levels, and downregulate Th2-type immune responses, ultimately enhancing atopic dermatitis outcomes (319).

#### 4.6.3 Anti-inflammatory effects in food allergy

Food allergy is a potentially life-threatening condition, triggered by IgE-mediated mast cell activation. These findings suggest that although mast cells are central to the classical allergic reaction pathway, other immune cells, like basophils and neutrophils, may also contribute. A range of flavonoids has demonstrated efficacy in mitigating food allergy. Formononetin, an isoflavone, not only inhibits degranulation of mast cells and basophils but also directly suppresses IgE production by human peripheral blood mononuclear cells through modulation of the JAK/STAT/PI3K-Akt signaling pathway, thereby attenuating allergic responses (127). In an ovalbumin-induced murine model of food allergy, nevadensin alleviates food allergy symptoms by inhibiting the c-Kit receptor, reducing the proliferation of bone marrow-derived mast cells, and promoting their apoptosis (320). Dihydromyricetin not only lowers the quantity of B cells and mast cells in the spleens of ovalbumin-allergic mice but also increases the population of Tregs, thereby ameliorating food hypersensitivity (321). Emerging evidence highlights the interplay between flavonoids, the immune system, and gut health as a promising avenue for alleviating allergic diseases. A comparative study has demonstrated that neohesperidin dihydrochalcone (NHDC), an artificially structurally modified flavonoid, exhibits superior efficacy in mitigating ovalbumin-induced food allergy in mice compared to its precursor compound, neohesperidin (NH). NHDC is more effective in restoring Th1/Th2 immune homeostasis and suppressing NOTCH/NF-κB signaling activation in the spleen. Both compounds can positively regulate the gut microbiota by enhancing the abundance of probiotic bacteria such as Lactobacillus, therefore ameliorating allergic symptoms. These findings underscore the potential of structural modifications to significantly enhance the anti-allergic properties of flavonoids (322).

## 5 Strategies to enhance the bioavailability of flavonoids

Although the immunoregulatory and anti-inflammatory properties of flavonoids have been demonstrated through *in vitro* and *in vivo* experiments as well as clinical studies, their therapeutic potential is constrained by low bioavailability. A major contributing factor is the low water solubility and limited membrane permeability exhibited by many flavonoids, which result in minimal gastrointestinal absorption (323). Fortunately, emerging technologies offer strategies to overcome these pharmacokinetic barriers, improving the clinical prospects of flavonoid therapies.

### 5.1 Nanotechnology

Studies have shown that nanotechnology and nanomaterials have successfully enhanced the water solubility and absorption of flavonoids. A notable example is naringenin, a hydrophobic flavonoid with limited aqueous solubility and stability. Electrospun pullulan nanofibers have been shown to rapidly disintegrate in artificial saliva and completely dissolve in water. When loaded with naringenin and sulfobutylether-β-cyclodextrin, these nanofibers significantly improve the aqueous solubility of naringenin, thereby enhancing its potential for oral absorption (324). The porous structure and plasticity exhibited by nanomaterials facilitate precise modulation of drug release profiles, enabling sustained, targeted, and stimuli-responsive delivery in therapeutic settings. Specially designed nanomaterials can effectively encapsulate poorly water-soluble drugs and regulate their release rate. In a mouse model of osteoarthritis, MXene-quercetin demonstrated superior effects in protective cartilage compared to free quercetin, highlighting the therapeutic advantages of nanodelivery systems (325). Topical drug delivery relies on passive diffusion, and prolonged drug retention helps improve efficacy. Nanocrystals can improve drug solubility and skin adhesion. Rutin nanocrystal gel exhibits more than 3 times higher permeability than conventional rutin gel, highlighting the substantial improvement enabled by nanotechnology (326). Nanomaterials can also help change the gastrointestinal absorption pathways of flavonoids. Lipid−based nanoparticles can encapsulate flavonoids and promote their integration into chylomicrons in the intestinal tract. These chylomicrons are then transported to the lymphatic vessels rather than the portal vein, thereby avoiding first−pass hepatic metabolism. This route helps increase systemic exposure and enhances the oral bioavailability of flavonoids (327).

### 5.2 Liposome delivery

Phospholipids possess both hydrophilic and hydrophobic domains, enabling them to effectively encapsulate poorly water-soluble flavonoids. Moreover, the phospholipid bilayer membrane of liposomes exhibits high biocompatibility and membrane fluidity, making them helping to promote biological permeability and nutrient absorption. At equivalent dosing, liposomal hesperidin demonstrates superior therapeutic efficacy compared with the unformulated compound (328).

### 5.3 Chemical modification

Chemical modification can significantly improve the water solubility and stability of drugs, thereby improving their bioavailability. Glycosylation and methylation modification of flavonoids have emerged as promising strategies to overcome pharmacokinetic limitations. Rutin has exhibited diverse pharmacological activities, but its clinical utility is constrained by poor water solubility and low bioavailability. These limitations can be improved through glycosylation modification. In animal studies, glycosylated rutin demonstrated stronger hepatoprotective effects than the unmodified compound (329). Fisetin also faces challenges, including insufficient stability, low oral bioavailability, and poor absorption. Biocatalytic methylation using engineered methyltransferases enables efficient modification of fisetin, leading to improved solubility, chemical stability, and lipophilicity, thereby enhancing its development and application (330).

### 5.4 Adding cosolvents

The addition of cosolvents is one of the most effective strategies to promote the solubility of non-polar drug molecules. Cosolvents such as ethanol and propylene glycol can efficiently extract flavonoids from foods, fruits, or herbs and enhance the solubility of flavonoids in the digestive system, thereby increasing their bioavailability. Flavonoids dissolved in ethanol can be easily formulated into oral or injectable preparations, facilitating the realization of their therapeutic potential (331).

## 6 Discussion

**Figure 1:**
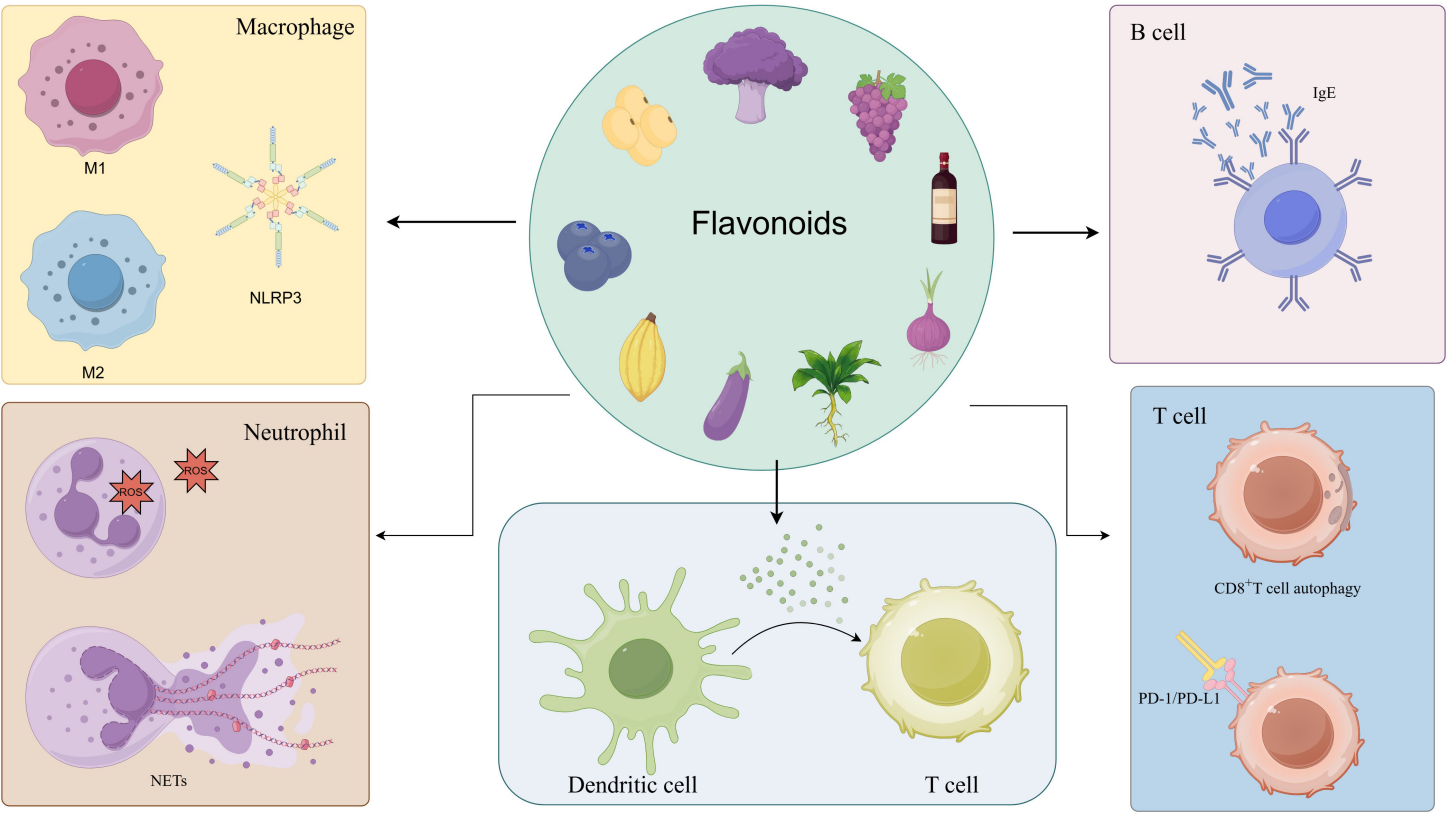
Flavonoids’ regulatory effects on key immune cells. Flavonoids can regulate immune cells including macrophages, dendritic cells, neutrophils, T cells, and B cells: inhibiting the polarization of macrophages toward the pro-inflammatory M1 phenotype and facilitating their conversion to the anti-inflammatory M2 type; reducing neutrophil activation and NET formation; modulating dendritic cell maturation and antigen presentation; influencing T cell differentiation, particularly by modulating Th17/Treg balance; and suppressing B cell activation and autoantibody production.

**Figure 2:**
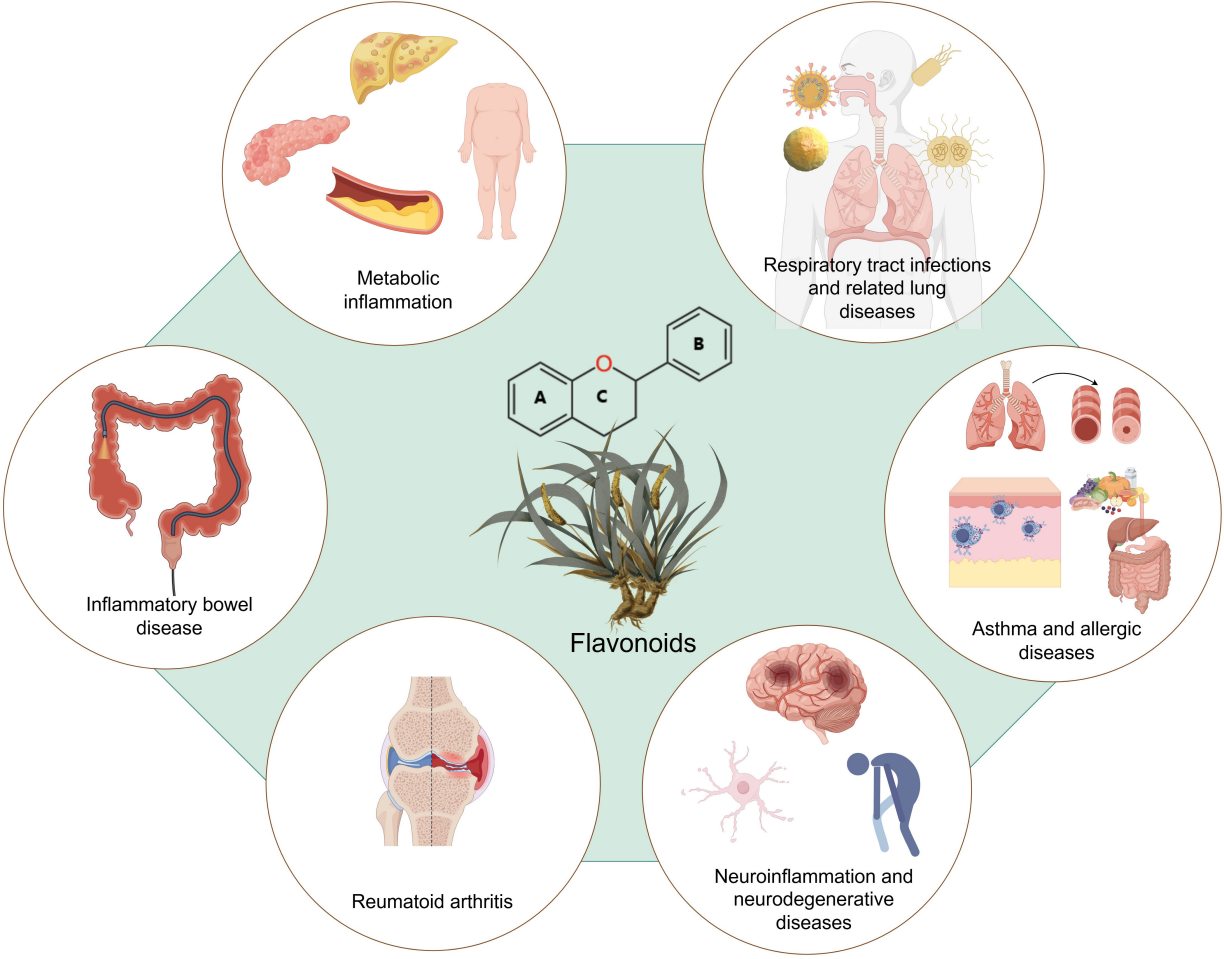
Flavonoids’ therapeutic applications in inflammatory diseases. Flavonoids show their therapeutic potential in inflammatory diseases, including inflammatory bowel disease, rheumatoid arthritis, metabolic inflammatory disorders, neuroinflammation and neurodegenerative diseases, respiratory tract infections and related lung diseases, asthma and allergic diseases.

This review provides a comprehensive overview of the regulatory effects of flavonoids on immune cell functions and their strategic applications in the management of inflammatory disorders (**Figures 1** and **2**). Studies have indicated that flavonoids regulate immune cell functioning through multi-target and multi-pathway mechanisms, exhibiting broad anti-inflammatory and immunomodulatory properties across various inflammatory conditions. Structurally, the C6-C3-C6 backbone serves as the fundamental scaffold for the biological activities of flavonoids, while different subclasses, including flavones, flavonols, flavanones, and isoflavones, exhibit distinct functional characteristics due to variations in their molecular structures.

With respect to immune regulation, flavonoids exhibit substantial regulatory effects on both innate immune cells (macrophages, neutrophils, and dendritic cells) and adaptive immune cells (T cells and B cells), highlighting their therapeutic relevance in immune-mediated inflammatory diseases. In intestinal inflammatory diseases, they contribute to the reduction of gut inflammation by preserving intestinal barrier integrity and modulating gut microbiota composition. In RA, they suppress synovial inflammation and prevent bone erosion. In metabolic diseases, they improve insulin sensitivity and mitigate adipose tissue inflammation, thereby alleviating metabolic dysregulation. However, their *in vivo* efficacy is often constrained by their low bioavailability, influenced by physicochemical characteristics, intestinal absorption, hepatic metabolism, gut microbiota composition, etc. To overcome the challenge of limited bioavailability, emerging strategies such as nanodelivery systems, structural modifications, and prodrug formulations are being actively investigated to enhance the pharmacokinetic properties and clinical utility. Future studies could focus on optimizing the molecular structure of flavonoids, which may yield structurally modified derivatives with enhanced pharmacological potency. For inflammatory diseases such as pulmonary disorders, developing inhalable formulations may achieve higher drug concentrations at target sites. Furthermore, novel drug delivery systems, such as pH-responsive carriers and yeast encapsulation, offer promising strategies to enhance the permeability and intestinal absorption of flavonoids.

Despite considerable advancements in the investigation of the immunomodulatory properties of flavonoids, notable limitations persist in the current research. Firstly, the majority of studies have been conducted using *in vitro* cell models or animal models, with a lack of clinical investigations, particularly high-quality RCTs. As flavonoids are natural compounds, they face significant challenges in securing patent protection, which in turn discourages commercial investment and limits the feasibility of conducting large-scale RCTs. Existing clinical studies often suffer from single-center experiments, small sample sizes, short follow-up durations, and the absence of standardized endpoint measures, which challenge comparative analyses across studies and the establishment of conclusive evidence. Dietary prevalence of flavonoids may introduce confounding factors and compromise the internal validity of clinical studies. These limitations collectively undermine the robustness of current clinical evidence. Future clinical research on flavonoids should prioritize larger sample sizes, longer follow-up durations, and multi-center study designs to enhance statistical power and generalizability. Moreover, investigating the differential therapeutic responses to flavonoids across diverse racial and population groups warrants further exploration to support personalized treatment.

Secondly, the deficiencies in the research approach should not be ignored. The concentrations of flavonoids employed in *in vitro* research sometimes exceed those attainable *in vivo*. Moreover, variations in dosing regimens, administration routes, and animal models across studies contribute to inconsistencies and limit the comparability of findings. For example, in quercetin treatment for RA in mice, Shen et al. (332) administered quercetin orally using normal saline containing 0.5% sodium carboxymethyl cellulose as solvent, with a maximum concentration of 100 mg/kg/day. While Yuan et al. (194) used intraperitoneal injection of a quercetin solution without reporting solvent, concentration, or dosage used. Most flavonoids exhibit poor water solubility, necessitating the use of cosolvents and solvents in experimental settings. However, the choice of such cosolvents and solvents can significantly influence the effective bioavailable concentration of the compound, thereby affecting pharmacokinetic and pharmacodynamic outcomes. Therefore, we recommend that studies involving flavonoids clearly report details of the administration protocol, including the compound concentration, the specific cosolvent and solvent used, the fasting status of experimental subjects, as well as the dosing frequency and route of administration. For studies employing multi-component formulations, the concentrations of individual flavonoids within the mixture should also be explicitly specified. Standardized reporting would enhance the reproducibility and facilitate robust meta-analyses to evaluate the therapeutic efficacy of flavonoids. Furthermore, *in vitro* studies that aim to simulate oral drug exposure should verify whether the applied flavonoid concentrations are achievable through *in vivo* metabolism following oral intake.

Third, the investigation of molecular processes remains insufficient. While numerous signaling pathways regulated by flavonoids have been identified, investigations into broader regulatory networks, such as epigenetic regulation and immune metabolic reprogramming, are still in their infancy. Future research could focus on investigating the crosstalk among signaling pathways modulated by flavonoids, which would significantly enhance our understanding of their complex regulatory mechanisms. The potential of flavonoids as modulators of epigenetic regulators, such as TET2, is also worth investigating. Moreover, there is a lack of comprehensive pharmacokinetics and safety data for flavonoids. The absence of standardized analytical methodologies has led to considerable variability in reported bioavailability values across studies. Long-term safety evaluations of flavonoid supplementation remain insufficient, particularly for vulnerable populations such as pregnant women, children, and the elderly. Moreover, research on potential interactions between flavonoids and conventional pharmaceuticals is limited, further contributing to uncertainties regarding their clinical application. The potential for flavonoids to enhance metabolic effects through combination therapy with established pharmacological agents, such as statins, ACEI/ARB, and SGLT2/GLP-1 receptor agonists, warrants systematic investigation. Exploring synergistic interactions between flavonoids and these drug classes could reveal novel therapeutic strategies with improved efficacy.

Mechanistically, flavonoids act by concurrently modulating multiple interconnected signaling pathways, reshaping the immune network in a mild yet sustained manner rather than strongly suppressing a single pathway. This characteristic renders flavonoids particularly suitable as immunomodulatory agents for chronic inflammatory disorders, especially for long-term maintenance therapy during the early or remission phases of the disease. Future research should conceptualize flavonoids as “multi-target network regulators” rather than conventional single-target drugs and prioritize investigations into their complex mechanisms. As a broad-spectrum and multi-target therapeutic agent, flavonoids require more comprehensive and rigorous evaluation of their anti-inflammatory effects to prevent the risk of excessive immunosuppression, which may lead to increased susceptibility to infections and malignancies.

## 7 Conclusion

Flavonoids have demonstrated broad immunoregulatory and anti-inflammatory effects in both innate and adaptive immune cells, with evidence supporting therapeutic potential in RA, intestinal inflammatory diseases, metabolic disorders, CVD, respiratory diseases, etc. Their multi-target, network-based mechanisms appear to contribute to sustained immune regulation, indicating potential suitability for long-term management of chronic inflammatory diseases. However, key translational challenges remain, including poor bioavailability, methodological inconsistencies, and a scarcity of high-quality clinical evidence. To better understand the clinical utility of flavonoids and assess their potential as adjunctive therapies, the following areas need to be further investigated: structural optimization, drug targeting, and inhalation delivery systems, standardized research reporting, and multi-omics data integration, along with rigorous multicenter clinical trials and comprehensive safety evaluations.
